# Characterization of Argonaute-containing protein complexes in *Leishmania*-infected human macrophages

**DOI:** 10.1371/journal.pone.0303686

**Published:** 2024-05-23

**Authors:** Atieh Moradimotlagh, Harsimran Kaur Brar, Stella Chen, Kyung-Mee Moon, Leonard J. Foster, Neil Reiner, Devki Nandan

**Affiliations:** 1 Department of Medicine, Division of Infectious Diseases, University of British Columbia, Vancouver, B.C, Canada; 2 Department of Biochemistry and Molecular Biology, Michael Smith Laboratories, University of British Columbia, Vancouver, B.C, Canada; Cochin Institute: Institut Cochin, FRANCE

## Abstract

The intracellular protozoan parasite *Leishmania* causes leishmaniasis in humans, leading to serious illness and death in tropical and subtropical areas worldwide. Unfortunately, due to the unavailability of approved vaccines for humans and the limited efficacy of available drugs, leishmaniasis is on the rise. A comprehensive understanding of host-pathogen interactions at the molecular level could pave the way to counter leishmaniasis. There is growing evidence that several intracellular pathogens target RNA interference (RNAi) pathways in host cells to facilitate their persistence. The core elements of the RNAi system are complexes of Argonaute (Ago) proteins with small non-coding RNAs, also known as RNA-induced silencing complexes (RISCs). Recently, we have shown that *Leishmania* modulates Ago1 protein of host macrophages for its survival. In this study, we biochemically characterize the Ago proteins’ interactome in *Leishmania-*infected macrophages compared to non-infected cells. For this, a quantitative proteomic approach using stable isotope labelling by amino acids in cell culture (SILAC) was employed, followed by purification of host Ago-complexes using a short TNRC6 protein-derived peptide fused to glutathione S-transferase beads as an affinity matrix. Proteomic-based detailed biochemical analysis revealed *Leishmania* modulated host macrophage RISC composition during infection. This analysis identified 51 Ago-interacting proteins with a broad range of biological activities. Strikingly, *Leishmania* proteins were detected as part of host Ago-containing complexes in infected cells. Our results present the first report of comprehensive quantitative proteomics of Ago-containing complexes isolated from *Leishmania-*infected macrophages and suggest targeting the effector complex of host RNAi machinery. Additionally, these results expand knowledge of RISC in the context of host-pathogen interactions in parasitology in general.

## Introduction

The leishmaniasis represents a broad spectrum of diseases caused by infection with protozoan parasite *Leishmania*. Leishmaniasis has several manifestations, including visceral, cutaneous or mucocutaneous symptoms [1]. Visceral leishmaniasis, mainly caused by *Leishmania donovani*, can be life-threatening when left untreated [2] and *L*. *donovani* is the model organism of this study. Unfortunately, the lack of effective drugs, toxic side effects, drug resistance and unavailability of approved human vaccines resulted in a rise in the incidence of leishmaniasis [1]. The *Leishmania* life cycle alternates between two distinct morphological stages: promastigote, the motile form found in the gut lumen of infected sand flies, and amastigote, the stationary form found in mononuclear phagocytes of the mammalian hosts [3]. Macrophages are the primary resident cells for *Leishmania*, and once the amastigote stage is established in the macrophages, the parasite grows and divide, result into chronic infection [4]. Due to the rise in the incidence of leishmaniasis worldwide, new strategies to combat and stop leishmaniasis are urgently needed. Elaborating our understanding of the interaction between the parasite and the host’s biology is critical in creating a comprehensive strategy to improve leishmaniasis treatment and combat the disease.

It is increasingly evident that non-coding RNAs (ncRNAs), particularly small ncRNAs (sncRNAs), play pivotal roles in numerous biological processes and diseases [5–8]. Notably, microRNAs (miRNAs) and tRNA-derived RNA fragments (tRFs) have been established as significant players in microbial infections [9–12]. Non-coding RNAs belong to a class of transcripts which do not translate into proteins [13]. The ENCODE project published that over 90% of all mammalian transcripts are ncRNAs [14,15]. The functional mechanism of sncRNAs is associated with Argonaute (Ago) proteins. These Ago proteins are highly conserved components of RNA interference (RNAi) pathways [16,17]. In humans, there are eight Ago family members: four belonging to the Ago clade, referred to as Ago1-4 proteins, which are ubiquitously expressed, and four belonging to the PIWI clade, namely PIWI1-4, primarily found in the germline [18]. Out of four mammalian Ago proteins, only Ago2 has endonuclease activity. To regulate target gene expression, sncRNAs and Ago proteins need to assemble to form RISC. The loading of sncRNAs onto Ago proteins is a complex and dynamic process mediated by the chaperones, including HSPs [19,20]. There is some evidence for selective sorting of particular small RNAs into specific Ago proteins. For example, novel sncRNAs appear to be selectively loaded onto mammalian Ago1 protein, as opposed to Ago2 protein, in cells infected with the Epstein-Barr virus (EBV) [21]. A similar sorting mechanism for distinct sncRNAs duplexes into RISCs with specific Ago proteins has been observed in lower organisms [22].

In RISC, Ago proteins are directed to target mRNAs with sequences that partially or fully complement those of the guide sncRNAs, and silence the targets’ expression by slicing them or by recruiting proteins that mediate translational repression and/or destabilization, depending on the guide-target complementarity. A majority of animal sncRNAs, such as miRNAs, generally repress target mRNAs not by slicing but by translational repression of the target mRNA [23]. In most situations where Ago2 is not involved in RNAi, TNRC6/GW182 protein (a scaffold protein) interacts with Ago proteins via its Ago binding domain and also recruits key components of repressor complexes, leading to inhibition of translation by deadenylation, decapping, and exonucleolytic degradation of the target mRNA [23,24]. It has been shown that the GW182 proteins have an increased affinity for Ago proteins loaded with guide RNAs compared to unloaded Ago proteins [25]. Thus, GW182 is recruited to Ago proteins once they are loaded with guide RNA and ready for target mRNA recognition [25]. This ensures that only mature RISC would be recruited for silencing.

It is well established that *Leishmania-*macrophage interaction results in deactivation of macrophages [26]. However, the molecular mechanisms used by *Leishmania* to deactivate its host is not fully understood. miRNAs play fundamental roles in macrophage activation, cytokine polarization, and inflammation resolution [27]. Thus, it is not surprising that *Leishmania* modulates macrophage miRNAs for its survival. In this context, a recent review addresses differential expression of miRNAs in each the parasite and the infected host and its implications for *Leishmania* survival and infectivity [28]. Recently, we have shown that *L*. *donovani* significantly increases the abundance of host macrophage c-Myc to promote its survival, and that this c-Myc upregulation is linked to downregulating host macrophage miRNAs. Deliberate c-Myc silencing reversed the *Leishmania*-mediated miRNA suppression and also reduced the intracellular survival of *Leishmania* [29]. Additionally, our recent findings revealed a high abundance of sncRNAs, such as tRFs and ribosomal RNA-derived fragments in exosomes released by both *L*. *donovani* and *L*. *braziliensis* [30]. These exosomes showed immunosuppressive effects in monocytes and dendritic cells [31]. More recently, we have shown that *Leishmania* infection increases the abundance of macrophage Ago1 protein along with levels of Ago1 in active Ago-complexes, suggesting the preferred use of Ago1 over other Ago proteins in RNAi in *Leishmania*-infected cells [32]. Further, using siRNAs to knockdown Ago1, we showed that expression of several previously reported *Leishmania* pathogenesis-related proteins was dependent on the level of host macrophage Ago1 [32]. The exploration of the roles of sncRNAs from both the host and the pathogen including *Leishmania* infections has begun to emerge [28,33,34]. Thus, the biology of sncRNAs and Ago proteins may contribute to *Leishmania* pathogenesis, unraveling a novel aspect of virulence that warrants further investigation. Since mature RISC is the ultimate core component of RNAi involved in gene regulation, it was of interest to investigate whether *Leishmania* influences host macrophage RISC composition to its advantage.

Since Ago proteins are the essential constituent of RISC, in this study, we biochemically characterized the proteome of Ago-complexes isolated from *Leishmania*-infected human macrophages and that of Ago-complexes from non-infected macrophages for comparison. We isolated Ago-complexes using a recently published biochemical isolation of Argonaute protein complexes by "Ago protein Affinity Purification by Peptide" (Ago-APP) [32,35] followed by liquid chromatography-mass spectrometry. SILAC-based quantitative proteomic analysis revealed that *Leishmania* modulated the expression of several host Ago-associated proteins in a qualitative and quantitative manner. Strikingly, *Leishmania* proteins were detected as constituents of host Ago-complexes in infected cells. Together, our results present the first report of comprehensive quantitative proteomics of Ago-complexes isolated from *Leishmania-*infected macrophages and suggest targeting core components of host RNAi machinery.

## Materials and methods

### Reagents and antibodies

THP-1 cells (TIB-202TM) were from ATCC. RPMI 1640 media and Fetal bovine serum (FBS) were purchased from Gibco. HEPES, penicillin/streptomycin, L-glutamine, Hanks’ balanced salt solution, M199 medium, folic acid, hemin, adenosine, Pepstatin A, Phorbol 12-myristate 13-acetate (PMA), PreScission enzyme (GE27-0843-01), and Amicon® Ultra-2 centrifugal filter devices (UFC200324) were purchased from Millipore Sigma. Anti-Ago1 (5053), anti-Ago2 (2897), anti-Ago3 (5054), anti-Lamin A/C (2032), anti-Calreticulin (12238), and anti-Calnexin (2679) were purchased from Cell Signaling. Anti-GST (SC-138), anti-HSC70 (HSP70) (SC-7298), and anti-Actin (SC-47778) were from Santa Cruz biotechnology. Anti-GAPDH (G041) was from Abm, and anti TRBP (Ab42018) was purchased from Abcam. RPMI 1640 media lacking L-glutamine, L-arginine, and L-lysine was purchased from Caisson labs. North2South Hybridization buffer (37549), North2South Hybridization Stringency Wash Buffer (37555), and Chemiluminescent Nucleic Acid Detection Module kit (89880) were purchased from ThermoScientific.

### Cultivation and differentiation of THP-1 cells

THP-1 cells, of a human leukemia monocytic cell line, were cultivated under conditions of 37°C and 5% CO_2_ in RPMI 1640 medium supplemented with 10% heat-inactivated FBS, 10 mM HEPES, 100 U/ml penicillin/streptomycin, and 2 mM L-glutamine. To induce differentiation, THP-1 cells were treated with 10 ng/ml PMA for 16–18 hours. Differentiated THP-1 (dTHP-1) cells were then subjected to washings with Hanks’ balanced salt solution (HBSS) and provided with fresh media without PMA. The cells were then rested for 24 hours before any subsequent treatment.

### SILAC labeling of THP-1 cells

For SILAC labeling of cells, RPMI 1640 media lacking L-glutamine, L-arginine, and L-lysine were supplemented with 10% heat-inactivated dialyzed FBS, 2 mM L-glutamine, 10 mM HEPES, and 100 U/ml penicillin/streptomycin. The SILAC medium was divided into two portions: the "light" RPMI was supplemented with normal isotopic abundance arginine (21 mg/l) and lysine (36.5 mg/l), while the "medium" RPMI was supplemented with ^13^C_6_-arginine (20.25 mg/l) and ^2^H_4_-lysine (37.5 mg/l). THP-1 cells were cultured in their respective SILAC media at 37°C and 5% CO_2_, for at least five passages, for adequate labelling.

### Subcellular fractionation

To prepare cytosolic fractions and endoplasmic reticulum-free nuclear fractions, a published protocol was followed [32,36]. In brief, dTHP-1 cells were lysed in hypotonic lysis buffer (HLB) (10 mM Tris-HCl, pH 7.5, 10 mM NaCl, 3 mM MgCl_2_, 0.3% NP-40, 1 mM NaF, 1 mM Na_3_VO_4_) supplemented with protease inhibitors and subjected to five passes through a 22-gauge needle to disrupt cells. Subsequently, the cells were centrifuged at 2,300 × g, and the resulting supernatant was collected as the cytoplasmic fraction. The nuclear pellet was washed and heated at 95°C in SDS-PAGE sample loading buffer and subjected to Western blotting.

### *Leishmania* culture and dTHP-1 cells infection

*Leishmania donovani*, Sudan strain 2S, was maintained as described before [32]. Promastigotes were cultured in M199 medium supplemented with 10% heat-inactivated FBS, 10 mM HEPES, 10 μg/ml folic acid, 3 μg/ml hemin, 2 mM L-glutamine, 100 U/ml penicillin/streptomycin, and 100 μM adenosine at 26°C. To infect dTHP-1 cells with parasites, stationary phase promastigotes were incubated with the cells at a MOI of 20:1. Infection rate determination was performed as described before [32].

### Preparation of GST (control) and GST-T6B affinity beads, and isolation of interacting proteins

Ago protein Affinity Purification by Peptides (Ago-APP) was performed as described previously [32,35]. Briefly, to prepare the affinity beads, glutathione-agarose beads (GE-HealthCare) were incubated with GST (control) or GST-T6B recombinant proteins for at least 3 hours. The total cell lysate of dTHP-1 cells was prepared in NET lysis buffer (50 mM Tris pH7.5, 150 mM NaCl, 5 mM EDTA, 0.5% NP-40, 10% glycerol, 1mM NaF) supplemented with protease inhibitors. The cytoplasmic fractions prepared in HLB were supplemented with EDTA (5mM) and DTT (0.5 mM), to optimize the buffer for Ago-APP procedure.

To isolate GST-T6B affinity beads-interacting proteins, the samples were separately incubated with glutathione-agarose beads coupled with GST (control) for 1 hour to remove proteins non-specifically interacting with GST. Non-bound materials were then incubated with the GST-T6B affinity beads to capture interacting proteins. The proteins bound to affinity beads were eluted either by heating beads with SDS-PAGE sample loading buffer at 95°C for 7 min or by PreScission enzyme cleavage. The samples eluted using PreScission enzymatic cleavage were concentrated using a centrifugal filter device with 3000 Dalton cut-off (Amicon), and then subjected to LC-MS/MS analysis.

### Isolation of GST-T6B interacting proteins from *Leishmania*

2×10^9^ stationary-phase *Leishmania* promastigotes were collected by centrifugation at 4°C. The pellet was washed twice with HBSS and once with TBS (10 mM Tris-HCl pH 7.4, 0.15M NaCl). Then the pellet was subjected to freeze/thaw twice to lyse *Leishmania*. Subsequently, the lysed *Leishmania* pellet was resuspended in 1 ml ice-cold NET lysis buffer supplemented with 1 mM DTT, EDTA-free protease inhibitor cocktail (Roche), 1 mM PMSF, and 5 μg/ml each of aprotinin, leupeptin and pepstatin A, incubated on ice for 20 min, and centrifuged at 23,000 × g for 20 min at 4°C. The supernatant was incubated for 1 hour with GST (control) beads to remove non-specific binding proteins. The resulting precleared elute was incubated with GST-T6B affinity beads to isolate interacting proteins as described in the section above.

### GST-T6B pull-down of RNA from dTHP-1 cells

Whole cell lysate of dTHP-1 cells was used for the isolation of Ago-interacting RNA using GST-T6B beads as described previously [35] with minor modifications. Briefly, post Ago-APP, 10% of the glutathione-agarose beads were removed for Western blotting analysis for Ago1 and Ago2 proteins. The remaining 90% of the beads were subjected to RNA isolation. Beads were incubated with 200 μl of Proteinase K buffer (200 mM Tris-HCl pH 7.5, 300 mM NaCl, 25 mM EDTA, 2% (w/v) SDS) containing 0.16 mg/ml proteinase K at 65°C for 15 min. Treated beads were removed by centrifugation at 2,650 × g for 2 min. To the resulting supernatant, 200 μl of Phenol: Chloroform: Isoamyl alcohol (25:24:1) was added, mixed vigorously, and kept at room temperature (RT) for 15 min, and then centrifuged at 15,300 × g for 15 min. The aqueous layer containing RNA was precipitated overnight by adding equal amount of isopropanol and 30 μg/ml of glycogen. The resulting RNA pellet was washed with cold 70% ethanol and subsequently dissolved in DEPC treated water.

### Northern blotting

The GST-T6B pulled-down RNAs were subjected to Northern blotting as described herein. Prior to loading of RNA onto the gel, an equal volume of 2X RNA loading dye (5mM EDTA, 0.01% bromophenol blue and 95% formamide) was added to the RNA sample. It was then heated at 70°C for 5 min and snap cooled on ice as described [37].

Prior to sample loading, the 15% denaturing polyacrylamide TBE/7M urea gel was pre-run in TBE at 100 V for at least 10 minutes [38]. Roughly, 0.9–3 μg of RNA was loaded onto the gel and subjected to electrophoresis followed by transferring to positively charged Hybond N+ nylon membrane at 10 V for 90 min at 4°C. Upon completion of the transfer, the membrane was UV auto cross-linked using a UV Stratalinker 2400 (Stratagene), and then dried at 50°C for 45 min. Next, the membrane was pre-hybridized in North2South Hybridization buffer on a shaker at 55°C for 30 min. Overnight (18–20 hours) hybridization was performed in the same pre-hybridization buffer with the addition of a biotinylated probe for let7a-5p miRNA at a concentration of 150 ng/ml. The sequence of a specific let7a-5p probe (5′-/Biosg/AACTATACAACCTACTACCTCA-3’) synthesized by IDT was based on a published report [35]. North2South Hybridization Stringency Wash Buffer (diluted to 1X) was used to wash blot three times for 15 min each, with agitation at 55°C. Further steps were carried out according to the instructions provided by the manufacturer of the Chemiluminescent Nucleic Acid Detection Module kit. Briefly, the membrane was blocked in supplied nucleic acid detection blocking buffer for 15 min with gentle shaking at RT followed by detection using Streptavidin: HRP conjugate (1:300) in the same buffer for 15 min at RT with agitation. Next, the membrane was transferred to a new container and washed four times for 5 min each with 1X wash buffer at RT with gentle shaking. The membrane was then transferred to a new container and incubated with Substrate Equilibration Buffer for 5 min at RT with gentle shaking. Subsequently, the membrane was incubated with the working solution, comprising equal volumes of luminol/enhancer solution and stable peroxide solution, and then exposed to Medical Blue X-ray film for signal capture.

### Liquid chromatography-tandem mass spectrometry (LC-MS/MS) and protein identification

For mass spectroscopy analysis, equal amounts of eluted and concentrated proteins from each treatment were mixed, reduced and alkylated [39], and run briefly on 10% resolving SDS-PAGE. The entire lane was excised and digested with MS-grade trypsin [40]. Resulting peptides were cleaned on C-18 STop And Go Extraction (STAGE) tips [41] using 40% (v/v) acetonitrile in 0.1% (v/v) formic acid as the elution buffer and analyzed on NanoDrop One (Thermo Scientific—A205, scopes) to approximate the concentration. To increase confidence, resulting samples were run on two different instruments, approximately 750 ng on Bruker TimsTof Pro and 600 ng on Bruker Impact II Qtof, coupled to either to nanoElute (Bruker Daltonik) or easy nLC 1200 (Thermo Scientific) nanoflow liquid chromatography system. Both instruments used Ionopticks’s Aurora series 25 cm x 75 μm C18 1.6 μm analytical column heated to 50°C with either 120 min or 90 min separation. Impact II Qtof analysis used acquisition settings as described [42]. Equivalent settings were used on TimsTof Pro analysis while TIMS mode was disabled.

Duplicate mass spectrometry (MS) runs were combined as fractions and searched on MaxQuant version 1.6.17.0 [43] against Uniprot’s human sequences (UP000005640), *Leishmania donovoni* BPK282A1, and common contaminant sequences. SILAC labels of medium arginine (^13^C_6_) and medium lysine (D_4_) were set for quantitation enabling iBAQ, requantify, and match-between-runs options. The data were filtered for 1% false discovery at protein, peptide and PSM levels. The mass spectrometry proteomics data have been deposited to the ProteomeXchange Consortium via the PRIDE [44] partner repository with the dataset identifier PXD037042. The reviewer account for the PRIDE data has the username reviewer_pxd037042@ebi.ac.uk.

### Bioinformatics analysis of mass spectrometry data

For LC-MS/MS data analysis, log_2_-transformed normalized ratios of Medium/Light (*Leishmania*-infected/ non-infected fold changes) were calculated using Microsoft Excel and R software (http://www.r-project.org). The one-sample T-test was utilized for the statistical comparison of log_2_-transformed normalized ratios against the hypothetical value of 0, indicating no fold change. The significance of each protein modulation was evaluated based on its distance from the origin, measured along axes (Manhattan distance) in a volcano plot of -log_10_ (p-value) versus log_2_(fold change). A higher Manhattan score indicates a greater significance in the modulation of the protein.

Gene Ontology (GO) annotation was done with the Uniprot Princeton GO Mapper. The specified parameters included setting the Organism (Annotation) to "Homo sapiens (GOA @EBI + Ensembl)" and the Ontology to "Generic slim." The proteins were categorized for two biological aspects: biological process and molecular function.

## Results

### Ago protein affinity purification by peptide to isolate macrophage Ago-complexes

**Fig 1:**
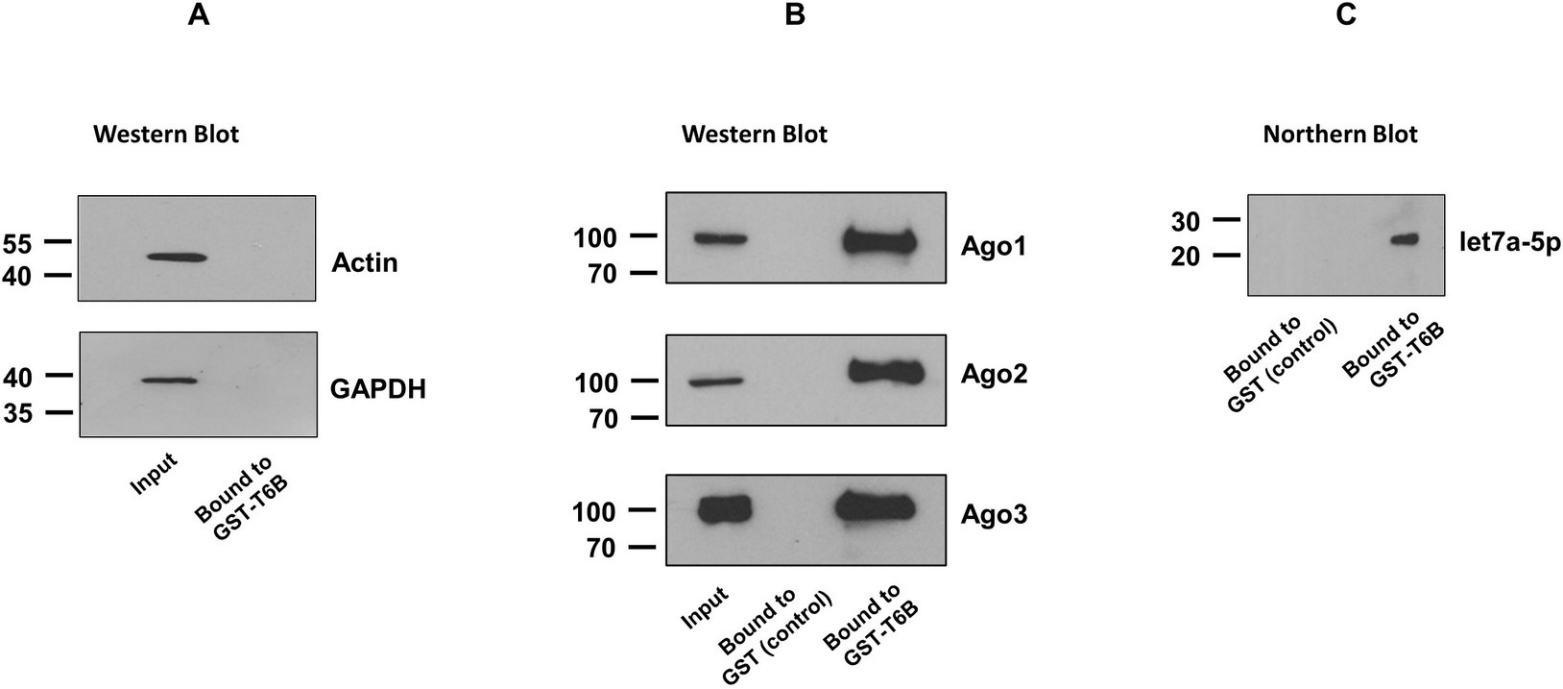
Isolation and validation of Ago-complexes from dTHP-1 cells using GST-T6B affinity beads. Recombinant GST (control) or GST-T6B were loaded on to glutathione-agarose beads. Whole cell lysate of dTHP-1 was incubated with GST (control) or GST-T6B separately and then the bound material was analyzed. **(A)** Actin and GAPDH were used as non-specific controls and analyzed to evaluate the specificity of the T6B affinity beads by Western blotting. **(B)** Ago1, Ago2 and Ago3 proteins’ presence was analyzed by Western blotting. **(C)** The presence of an abundant endogenous miRNA let7a-5p was tested through Northern blotting. The result shown represents one of the three independent experiments.

Our recent results raised the possibility that *Leishmania* hijacks macrophage Ago-complexes to promote its survival [32]. Hence, it was of interest to investigate whether *Leishmania* induces changes in the composition of Ago proteins complexes and their associated proteins qualitatively and/or quantitatively. To investigate this possibility, we used a published approach “Ago protein Affinity Purification by Peptides” (Ago-APP) to isolate Ago-complexes [32,35], which allows for the simultaneous isolation of all Ago proteins and their associated proteins from a variety of species and cell lines [35]. This powerful pull-down assay involves GST coupled with the T6B peptide, which is a part of the TNRC6/GW182 protein family and that efficiently interacts with all Ago proteins [35]. In fact, we have recently used Ago-APP to isolate active Ago1 protein as part of a complex from the cytosol of non-infected and *Leishmania-*infected dTHP-1 cells [32]. For the current study, we further validated Ago-APP by testing the presence of Ago3 in addition to Ago1 and Ago2. In this regard, GST (control) and GST-T6B peptide beads were prepared as described in “Materials and methods”. Ago-APP was performed using whole cell lysate of dTHP-1 cells as described previously [32]. The presence of proteins in the eluted material was investigated by Western blotting using appropriate antibodies. Actin and GAPDH were used as negative controls and as expected, they did not bind to GST-T6B (Fig 1A). As expected, Ago1, Ago2 and Ago3 were detected in material eluted from GST-T6B beads, and not in the material from GST (control) sample (Fig 1B), further validating the effectiveness of Ago-APP in the isolation of Ago proteins and possibly associated proteins. We also analyzed the expected association of sncRNAs such as miRNAs as part of active Ago-complexes by Northern blotting. Accordingly, bound materials from GST (control) and GST-T6B were investigated for the presence of endogenous miRNA let7a-5p. As expected, let7a-5p was pulled down by GST-T6B beads only, likely due to their binding to functional Ago-complexes (Fig 1C).

### Ago proteins are mainly restricted to the cytoplasm of macrophages

**Fig 2:**
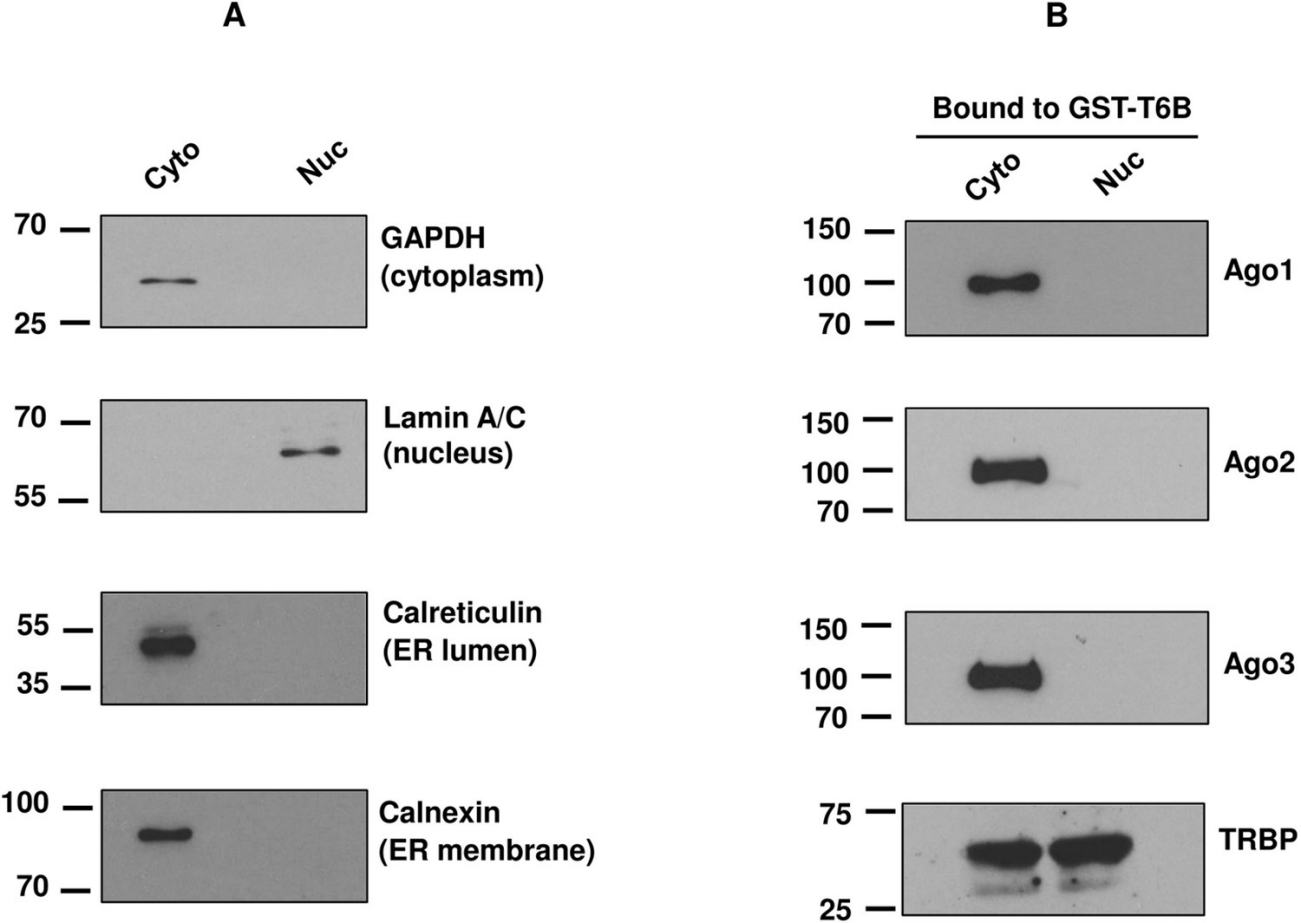
Subcellular localization of Ago proteins. **(A)** dTHP-1 cells were subjected to subcellular fractionation to yield cytoplasmic and ER-free nuclear fractions as described in “Materials and methods”. The purity of the cytoplasmic and ER-free nuclear fractions was evaluated by fraction-specific markers using Western blotting. **(B)** The cytoplasmic and nuclear fractions from dTHP-1 cells were separately incubated with GST-T6B affinity beads as described, in “Materials and methods”. The binding materials were analyzed by Western blotting using antibodies for Ago1, Ago2, Ago3, and TRBP. The presented data is representative of three separate experiments.

After validating the GST-T6B peptide affinity column for its interaction with Argonaute proteins from whole cell lysate, it was of interest to isolate Ago-complexes from the cytoplasm and the nucleus separately. In mammalian cells, RNAi is involved in post-transcriptional repression of gene expression, often in the cytoplasm, by RISC. However, several recent reports have shown the presence of Ago proteins in the nucleus, in addition to the cytoplasm [36,45]. Moreover, synthetic siRNAs have the potential to control nuclear gene expression, which suggests the existence of RISC factors in the nucleus. A published protocol [32,36] which was developed to produce nuclear fraction free from endoplasmic reticulum (ER) contamination was used for this study. This is important, as Ago proteins are known to be associated with the ER [46]. The ER is attached to the outer membrane of the nucleus. Calnexin (ER membrane marker) and Calreticulin (ER lumen marker) were used to test the potential contamination of ER in the nuclear fraction. As expected, GAPDH and Lamin A/C could only be detected in the cytosolic and nuclear fraction, respectively, while calreticulin and calnexin were restricted to cytoplasmic fractions, strongly suggesting the absence of ER in the nuclear fraction (Fig 2A).

In addition to investigating the presence of Ago1 and Ago2, we probed cell fractions for Ago3 and TRBP (transactivation response element RNA-binding protein). TRBP is an RNA binding protein which has been demonstrated to be part of RISC [47]. TRBP is known to exist in mammalian cell nuclei [36] and may play a role in transcription [48]. The cytoplasmic and ER-free nuclear fractions of dTHP-1 cells were incubated separately with GST-T6B to enrich Ago proteins as described in “Materials and methods”. Subsequently, the bound materials were investigated for the presence of proteins of interest using Western blotting. Fig 2B shows Ago1, Ago2 and Ago3 present in the cytoplasmic fraction, but undetectable in the nuclear fractions. However, TRBP could be detected in both cytoplasmic and nuclear fractions (Fig 2B). Taken together, our data strongly indicate the absence of Ago proteins in the nuclei of our model macrophages and hence only cytoplasmic fractions were used for subsequent studies. However, we cannot completely rule out the presence of non-detectible amount of Ago proteins in the nucleus.

### Proteomic analysis reveals *Leishmania-*mediated alterations in the protein composition of host Ago-containing complexes

We sought to investigate the possible modulation of cytoplasmic Ago-containing complexes in macrophages infected with *Leishmania*, with focus on their protein components. For a detailed characterization of the proteome of Ago-containing complexes, we used the GST-T6B affinity pull-down assay followed by SILAC-based quantitative mass spectrometry analysis as described in “Materials and methods” and shown in a schematic diagram (S1 Fig). For this analysis, cells grown in “Light” (L) medium were used as non-infected control, while cells grown in “Medium” (M) medium were infected with *Leishmania*. Cytoplasmic fractions were prepared from non-infected and *Leishmania*-infected cells and were each pre-cleared by incubation with GST (control) beads to remove non-specific interactions for GST-T6B affinity beads. Precleared cytoplasmic fractions were then incubated with GST-T6B affinity beads individually to capture Ago-complexes. Proteins bound to GST-T6B beads were released by enzymatic cleavage and concentrated. Equal amounts of proteins from each fraction were mixed and used for LC-MS/MS analysis. The method used is referred to as Mixing after Purification (MAP) SILAC where samples undergo affinity-based purification and are subsequently combined for mass spectrometry analysis.

Prior to LC-MS/MS analysis, a small aliquot from bound materials of three replicates was tested to confirm the presence of Ago1 through Western blotting. In addition, a partial aliquot of GST-T6B bound material from cytoplasmic fraction of non-infected and *Leishmania*-infected cells were subjected to SDS-PAGE followed by silver staining to detect the spectrum of proteins eluted using the Ago-APP (S2 Fig).

### Overview of identified proteins from Ago-complexes of macrophages

From the proteomics analysis, we identified 160 proteins. After filtering GST-T6B (bait)-related proteins (TNRC6B, and GSTP1), and Lamin-A/C (LMNA) protein as a contamination in cytoplasmic fractions, 157 proteins were specifically found in GST-T6B bound materials, including Ago1-3 proteins, as expected (S1 Table). Although we could not detect Ago4 in our preparations, could be due to very low abundance, the presence of other three Ago proteins validated the use of GST-T6B pull-down assay to isolate Ago-containing complexes.

We conducted characterization of Ago-interacting macrophage proteins, for those detected in at least two out of three biological replicates in both non-infected and *Leishmania*-infected macrophages. The results, presented in Table 1, show a comprehensive overview of 51 identified proteins interacting with Ago proteins.

**Table 1:** Ago-associated proteins in macrophages.

Protein name		Gene name	Number of detection (out of three replicates)
1	78 kDa glucose-regulated protein	HSPA5	3
2	Heat shock cognate 71 kDa protein	HSPA8	3
3	Heat shock 70 kDa protein 6	HSPA6	3
4	Elongation factor 1-gamma	EEF1G	3
5	Stress-70 protein, mitochondrial	HSPA9	3
6	Histone H2A.J	H2AFJ	3
7	GrpE protein homolog 1, mitochondrial	GRPEL1	3
8	Polyubiquitin-C	UBC	3
9	Protein argonaute-2	AGO2	3
10	Protein argonaute-1	AGO1	3
11	Tubulin beta chain	TUBB	3
12	E3 ubiquitin-protein ligase CHIP	STUB1	3
13	Elongation factor 1-beta	EEF1B2	3
14	Palmitoyl-protein thioesterase 1	PPT1	3
15	Elongation factor 1-alpha 1	EEF1A1	3
16	ATP synthase subunit alpha, mitochondrial	ATP5A1	3
17	Actin	ACTB	3
18	ATP synthase subunit beta, mitochondrial	ATP5B	3
19	Adenylyl cyclase-associated protein 1	CAP1	3
20	Heat shock protein HSP 90-beta	HSP90AB1	3
21	Endoplasmin	HSP90B1	2
22	Fatty acid-binding protein, epidermal	FABP5	2
23	Voltage-dependent anion-selective channel protein 1	VDAC1	2
24	Peroxiredoxin-1	PRDX1	2
25	Heat shock 70 kDa protein 1A	HSPA1A	2
26	Aminopeptidase N	ANPEP	2
27	LanC-like protein 1	LANCL1	2
28	Prelamin-A/C	LMNA	2
29	Calreticulin	CALR	2
30	Peptidyl-prolyl cis-trans isomerase B	PPIB	2
31	Protein disulfide-isomerase A3	PDIA3	2
32	Peroxisomal multifunctional enzyme type 2	HSD17B4	2
33	Histone H2B type 1-B	HIST1H2BB	2
34	Ras-related protein Rab-1A	RAB1A	2
35	Glyceraldehyde-3-phosphate dehydrogenase	GAPDH	2
36	Protein disulfide-isomerase A6	PDIA6	2
37	Lysosome-associated membrane glycoprotein 1	LAMP1	2
38	Ras-related protein Rab-7a	RAB7A	2
39	Dolichyl-diphosphooligosaccharide—protein glycosyltransferase subunit 1	RPN1	2
40	Ras-related C3 botulinum toxin substrate 2	RAC2	2
41	Transmembrane protein 33	TMEM33	2
42	Dolichyl-diphosphooligosaccharide—protein glycosyltransferase subunit 2	RPN2	2
43	Vesicle-trafficking protein SEC22b	SEC22B	2
44	Moesin	MSN	2
45	Dolichyl-diphosphooligosaccharide—protein glycosyltransferase 48 kDa subunit	DDOST	2
46	ADP/ATP translocase 3	SLC25A6	2
47	Protein disulfide-isomerase	P4HB	2
48	Integrin beta-2	ITGB2	2
49	ADP/ATP translocase 2	SLC25A5	2
50	60 kDa heat shock protein, mitochondrial	HSPD1	2
51	40S ribosomal protein S3	RPS3	2

**Fig 3:**
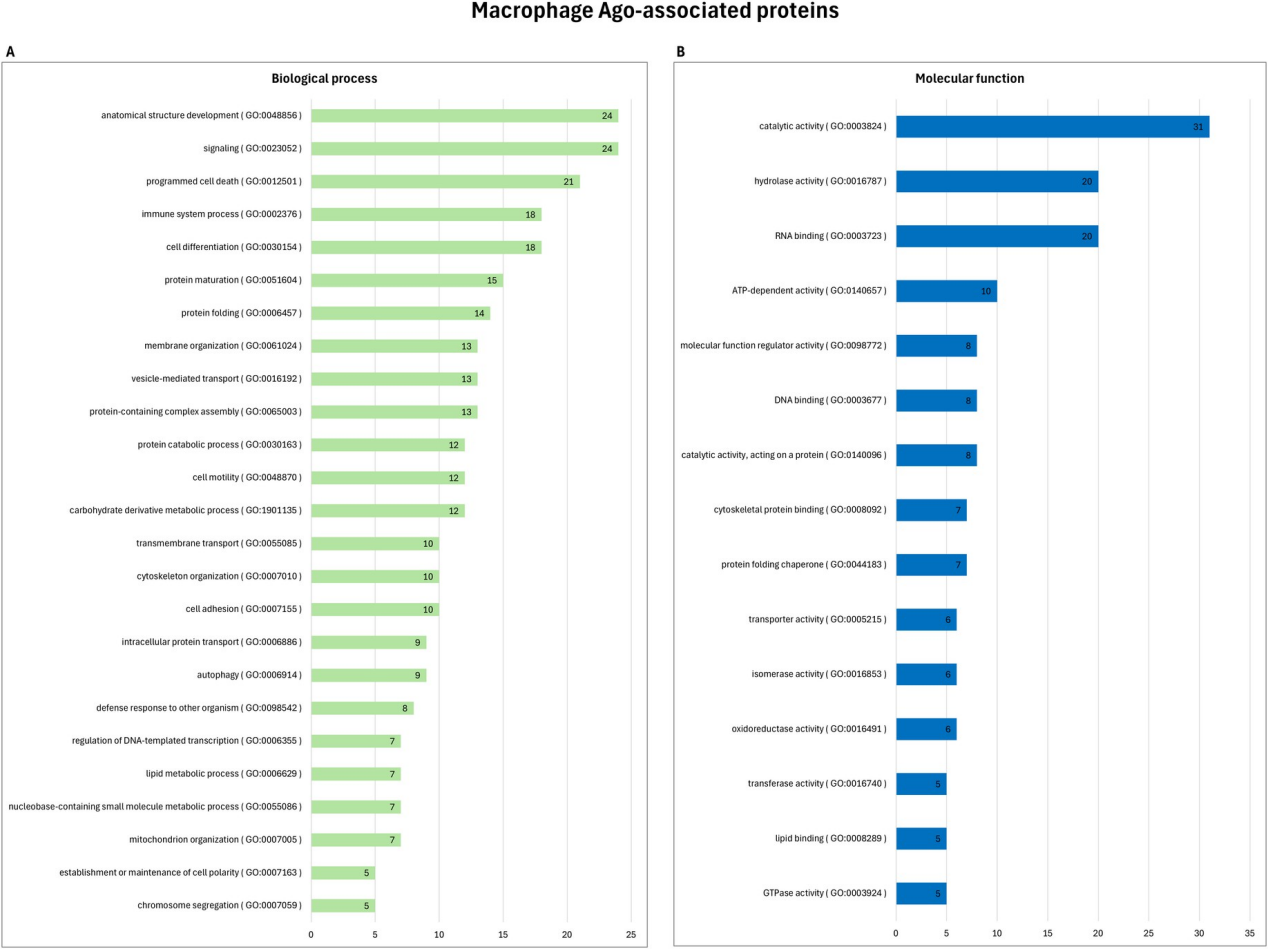
Gene ontology analysis of Ago-associated proteins in macrophages. The 51 proteins which were identified in at least two out of three replicates using Ago-APP were categorized according to **(A)** biological processes, or **(B)** molecular function, using Princeton University generic gene ontology (GO) term mapper. The bars represent the number of proteins associated with each GO term. GO terms categories that encompass less than five protein members (< 10%) were omitted.

These proteins were further subjected to Gene Ontology (GO) analysis, based on their known biological processes or molecular functions. Fig 3 provides a graphical representation of the distribution of these proteins in different categories. The majority of identified proteins’ molecular function include catalytic activity, hydrolase activity, RNA binding, ATP-dependent activity, molecular function regulator activity, DNA binding, catalytic activity, cytoskeletal protein binding, and protein folding chaperone. These findings suggest a diverse range of molecular functions associated with Ago-complexes in both non-infected and *Leishmania*-infected macrophages. The implications of these interactions shed light on the Ago-associated proteins in macrophages and the intricate molecular mechanisms involved in responding to *Leishmania* infection.

### Quantitative LC-MS/MS analysis of Ago-containing complexes

**Fig 4:**
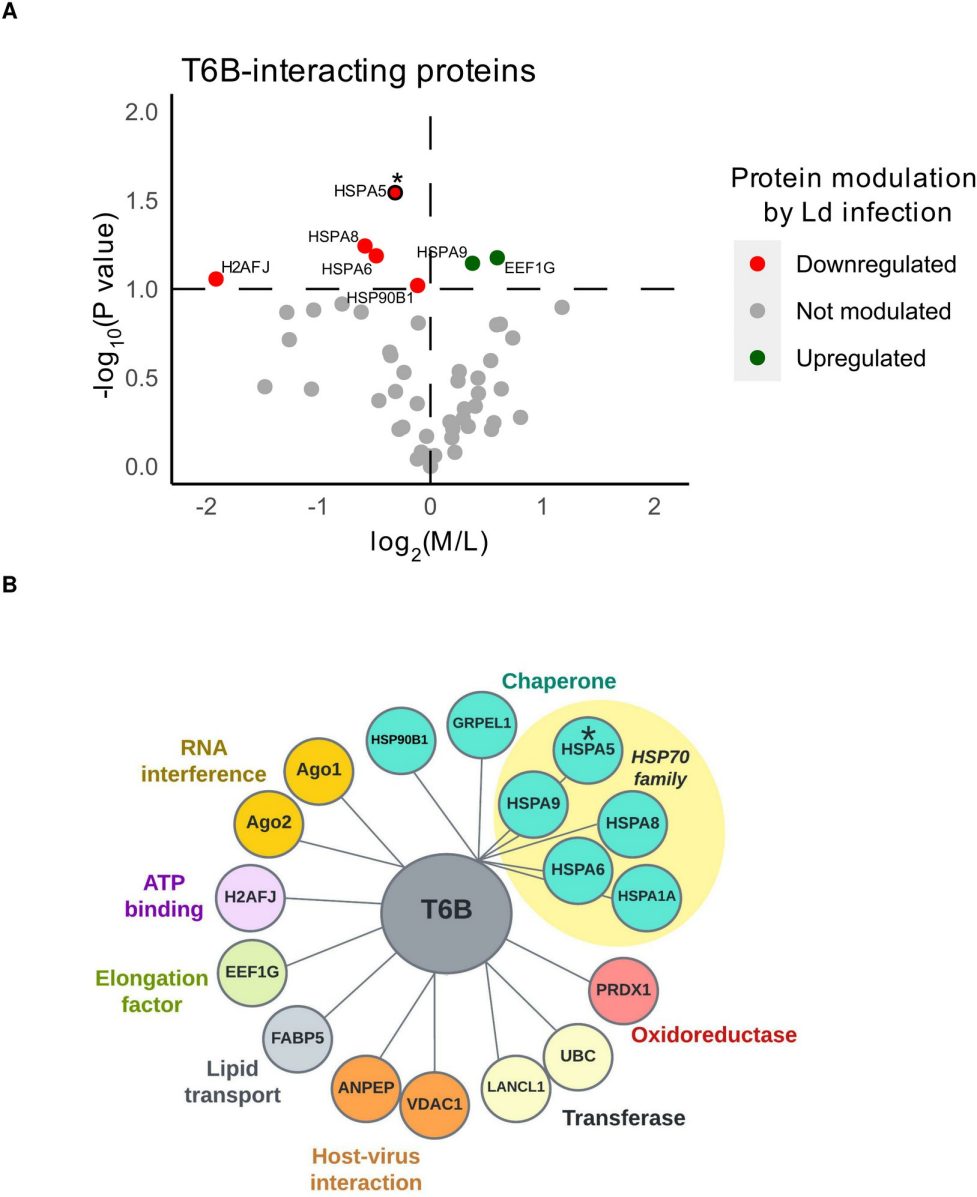
Comparative proteomics of cytosolic T6B-interacting proteins from non-infected and *Leishmania*-infected macrophages. **(A)** The volcano plot represents *Leishmania* infection-modulated host proteins with a p-value threshold of < 0.1, among the 51 detected proteins in at least two out of three experiments. * p-value < 0.05. L: Light (Non-infected), M: Medium (*Leishmania*-infected). **(B)** The bubble plot represents *Leishmania*-infection modulated host proteins with a p-value < 0.2, classified by their molecular and biological functions according to the UniProt database. * p-value < 0.05.

Quantitative ratios comparing protein levels bound to GST-T6B in *Leishmania*-infected versus non-infected cells were calculated for 51 proteins shown in Table 1. The abundance of 17 human proteins was differentially expressed between Ago-complexes obtained from non-infected and those from *Leishmania*-infected cells (Table 2). Amongst these modulated Ago-associated proteins, 11 were downregulated, and 6 were upregulated in infected cells compared to non-infected cells. Moreover, the T6B-interacting proteins most significantly modulated by *Leishmania* are predominantly heat shock proteins (HSPs), and the majority are downregulated (5 out of 6 HSPs) (Fig 4A).

**Table 2:** Most significant modulated Ago-associated proteins in *Leishmania*-infected cells, compared to non-infected cells.

Protein name	Gene name	Average modulation (log_2_)
Fatty acid-binding protein, epidermal	FABP5	-2.7206
Histone H2A	H2AFJ	-1.9146
Polyubiquitin-C	UBC	-1.2827
Peroxiredoxin-1	PRDX1	-1.0428
Voltage-dependent anion-selective channel protein 1	VDAC1	-0.7891
Heat shock 70 kDa protein 1	HSPA1A	-0.6186
Heat shock cognate 71 kDa protein	HSPA8	-0.5845
Heat shock 70 kDa protein 6	HSPA6	-0.4840
78 kDa glucose-regulated protein	HSPA5	-0.3149
Endoplasmin	HSP90B1	-0.1145
Aminopeptidase N	ANPEP	-0.1089
Stress-70 protein, mitochondrial	HSPA9	0.3753
Protein argonaute-1	AGO1	0.5910
Elongation factor 1-gamma	EEF1G	0.5966
Protein argonaute-2	AGO2	0.6239
LanC-like protein 1	LANCL1	0.7348
GrpE protein homolog 1, mitochondrial	GRPEL1	1.1751

**Fig 5:**
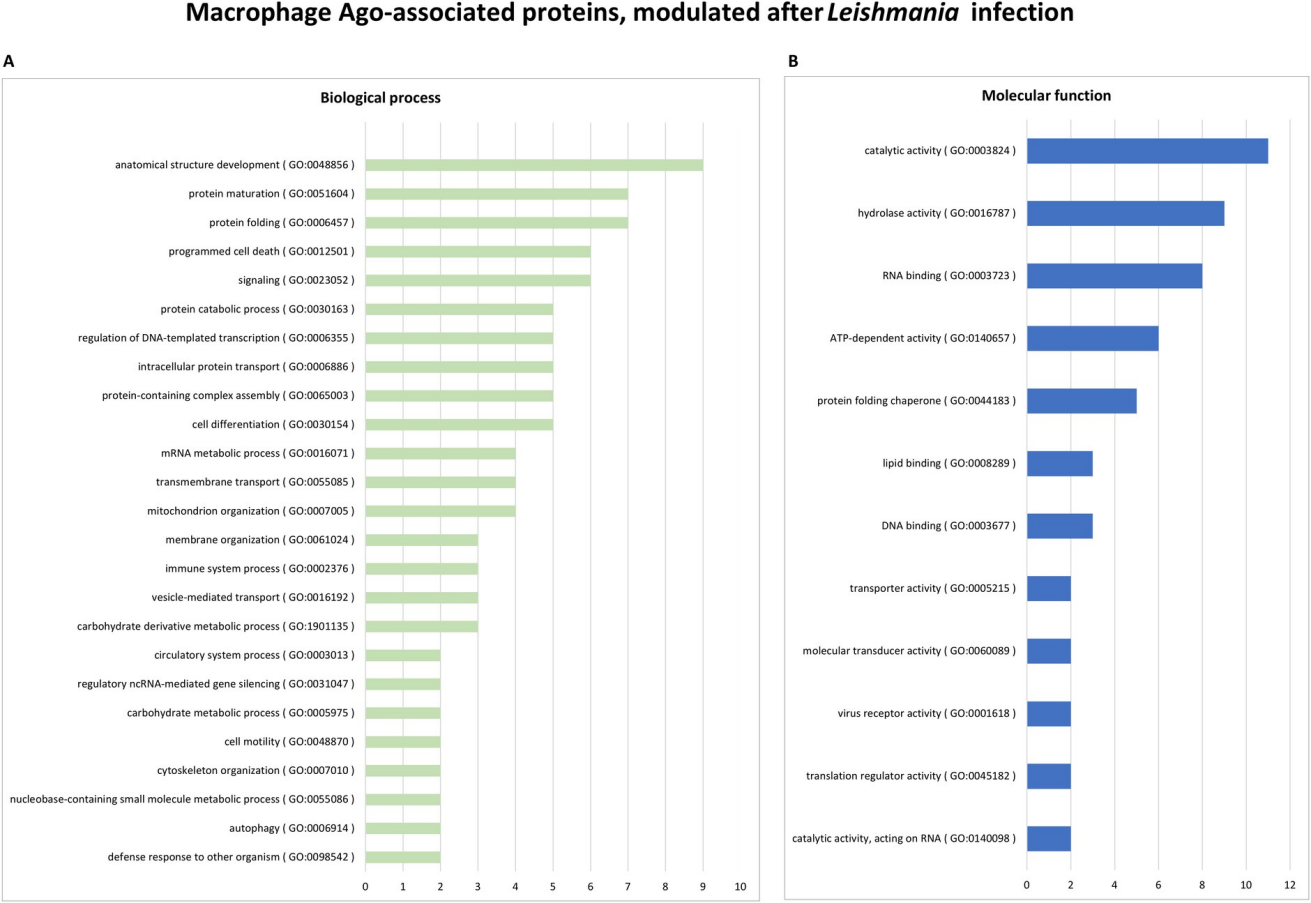
Gene ontology analysis of modulated Ago-associated proteins following *Leishmania* infection. Differentially expressed Ago-associated host proteins are categorized according to **(A)** biological processes and **(B)** molecular function, using Princeton University generic gene ontology (GO) term mapper. The bars represent the number of proteins associated with each GO term. GO terms categories that encompass only one protein member are omitted.

The gene ontology (GO) analysis was performed on the 17 differentially expressed proteins shown in Table 2. The result is presented as a bubble plot depicting macrophage cytosolic T6B-interacting proteins altered during *Leishmania* infection (Fig 4B). The most common biological process GO term was “chaperone”, which included HSP90B1, GRPEL1 and five proteins of the HSP70 family (7 out of 17) (Fig 4B). Host proteins involved in RNA interference, protein translation, ATP binding, transferases, oxidases, and host-virus interaction were also found to be modulated following *Leishmania* infection (Fig 4B). These differentially expressed proteins were also categorized according to their biological processes or molecular function, using Princeton University generic gene ontology term mapper (Fig 5).

### *Leishmania* proteins interact with host Ago-complexes

**Fig 6:**
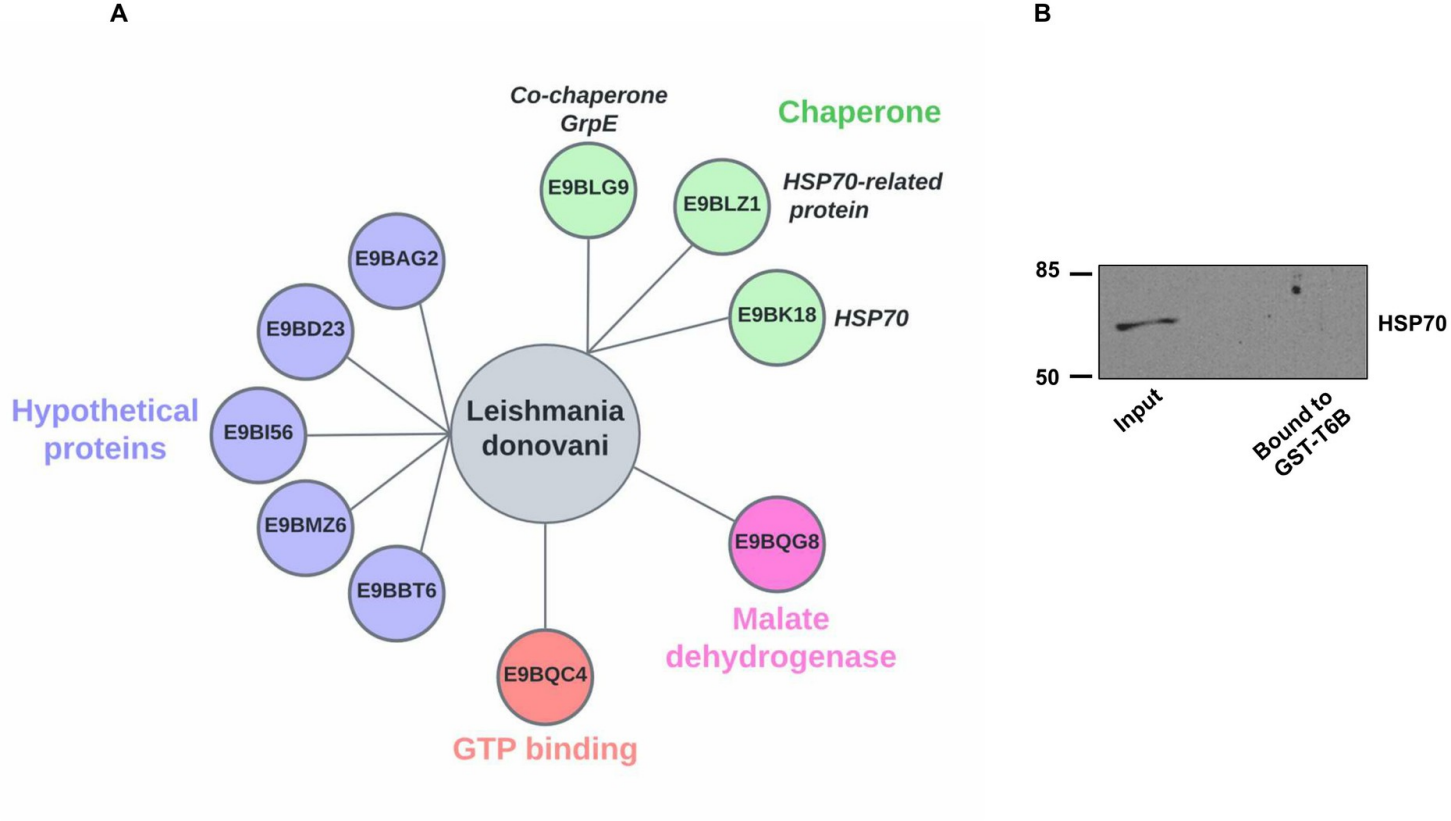
Ago-associated *Leishmania* proteins isolated from infected cells. **(A)** Bubble plot of *Leishmania* proteins identified in Ago-containing complexes eluted from Ago-APP performed using infected cells, classified by their functions. **(B) Selective binding of *Leishmania* HSP70 to GST-T6B.** Whole cell lysate of *L*. *donovani* (input material) was precleared with GST (control) beads followed by incubation with GST-T6B affinity beads. Non-bound materials were washed thoroughly, and the bound material was eluted and tested for the presence of HSP70 protein by Western blotting.

Strikingly, out of the total identified proteins, ten belonged to *L*. *donovani*, including HSP70, HSP70-related protein 1, Co-chaperone GrpE, and other proteins (Table 3, Fig 6A). We note that the identification of *Leishmania* proteins as constituents of Ago-containing complexes in infected cells is only qualitative, as parasite proteins were not labeled using SILAC media. To rule out the possibility that *Leishmania* proteins could directly interact with GST-T6B, rather than as part of Ago-complexes, we incubated *L*. *donovani* whole cell lysate with GST-T6B affinity beads. Bound proteins were tested for the presence *Leishmania* HSP70, the only *Leishmania* protein with an available antibody, in Western blotting. As shown in Fig 6B, no HSP70 signal was detected in the bound material. This strongly indicates that *L*. *donovani* HSP70 was isolated as a part of Ago-complexes, rather than through direct interaction with GST-T6B, and suggests that it may be incorporated into host Ago-complexes during *Leishmania* infection.

**Table 3:** Ago-associated *Leishmania* proteins.

Protein ID	Gene ID	Gene description
E9BLZ1	13385668	Heat shock 70-related protein 1, mitochondrial precursor, putative
E9BK18	13387132	Heat-shock protein hsp70, putative
E9BLG9	13385496	Co-chaperone GrpE, putative
E9BQG8	13392588	Malate dehydrogenase, putative
E9BQC4	13392415	Small GTP-binding protein Rab18, putative
E9BAG2	13389313	Hypothetical protein
E9BI56	13388877	Hypothetical protein
E9BD23	13393231	Hypothetical protein
E9BMZ6	13389645	Hypothetical protein
E9BBT6	13391409	Hypothetical protein

## Discussion

Recently, we have shown that *Leishmania* infection upregulated macrophage Ago1 level [32]. In addition, siRNA-mediated silencing of Ago1 in macrophages showed that expression of several previously reported *Leishmania* pathogenesis-related proteins was dependent on the level of macrophage Ago1 [32]. This raised the hypothesis that *Leishmania* could hijack host RNAi effector RISC complex for its advantage. Thus, the current study was conducted to test this hypothesis. A comprehensive quantitative proteomic analysis of Ago-complexes was performed on materials eluted from GST-T6B affinity pull-down assay which has the capability to isolate all human Ago proteins and their associated proteins [35]. It is known that unloaded Ago proteins are unstable and prone to proteasome degradation pathway [49,50] and it is likely that isolated Ago proteins and interacting proteins are part of the functional effector complex (RISC) of RNAi.

SILAC-based quantitative LC-MS/MS approach was used to identify the interactome of Ago proteins in *Leishmania*-infected and non-infected control host cells. SILAC-MS/MS-based technology offers outstanding sensitivity for protein detection and provides the capability for high-throughput, whole proteome analysis. This technology also allows mixing of samples at early stages to minimize variations due to technical error and offer the comparison of multiple investigational conditions in a single run.

The absence of Ago proteins in the nuclear fractions of non-infected and *Leishmania*-infected macrophages was unexpected. The nuclear role of Ago proteins has recently been summarized in a review by Bajczyk et al [51]. It has recently been shown that Ago1 binds to the promoter of actively transcribed genes in human cancer cells like a bona fide transcription factor [52]. The absence of Ago proteins in the nuclear fractions in our current study could be due to the low abundance of nuclear Ago proteins, which may require additional input material to enable detection by Western blotting/mass spectrometry assays. It is also possible that the role of Ago proteins is restricted to the cytoplasm in our model macrophage dTHP-1.

Since Ago proteins could not be detected in the nuclear fractions of non-infected and *Leishmania-*infected dTHP-1 cells (Fig 2B), our analysis was restricted to the cytoplasmic interactome of Ago proteins. This analysis identified 51 interactors common between infected and non-infected cells in at least two out of three replicates. Out of the 51 interactors, the levels of 17 human proteins were modulated in Ago-complexes obtained from *Leishmania*-infected compared to non-infected cells. The presence of Ago1, Ago2 and Ago3 validated the LC-MS/MS results. It was of interest to investigate whether any of these Ago-associated modulated proteins had previously been shown to have a role in *Leishmania* pathogenesis. For this, we compared our current data with the results obtained from our recent study investigating Ago1-dependent *Leishmania*-modulated proteins [32]. HSPA5, PRDX1, and EEF1G proteins were Ago1-dependent, found to be downregulated in *Leishmania*-infected cells in both studies, thus further emphasizing the importance of the results from our current study.

Interestingly, we also identified ten *L*. *donovani* proteins as constituents of Ago-complexes in infected cells. One can argue that these *Leishmania* proteins may have come from macrophage-mediated parasite degradation. Our previously published studies, where *Leishmania* parasite burden assays were conducted at an early and late time points of infection in dTHP-1 cells, found no significant change in parasite burden from 3 to 24 hours of infection, which suggests an insignificant level of parasite clearance [53,54]. In addition, the small number of *Leishmania* proteins identified in this study also argues against generalized *Leishmania* degradation. Thus, our data taken together suggests that generalized parasite clearance is unlikely a significant contributor to the *Leishmania* proteins detected in the Ago complexes.

Out of ten parasite proteins, five were hypothetical, and the functions of the remaining five in *Leishmania* pathogenesis were investigated. Out of these five, two were HSP70 and HSP70-related protein. It is known that *Leishmania* HSP70 is upregulated in infected macrophages. Moreover, infection with promastigotes overexpressing HSP70 could neutralize the host cells highly oxidative environment and renders the pathogen resistant [55,56]. Nevertheless, the role of parasite proteins identified in Ago complexes isolated from infected cells remains unclear. It will be of interest to determine the precise role of pathogen proteins as constituents of Ago complexes and their subsequent potential role in *Leishmania* pathogenesis. One approach to address this could be to generate *Leishmania* deficient in one or more pathogen proteins with known functions and use them in macrophage infection studies.

Notably, among 17 host modulated proteins in Ago-complexes, six proteins belonging to the host HSP family were mostly downregulated in infected cells compared to non-infected cells (Table 2, Fig 4A). This result is striking, as it is known that the Hsp70/Hsp90 multi-chaperone systems are involved in the ATP-dependent conformation change of Ago proteins to an open and active state [20]. This process allows them to accommodate the RNA duplex and thus is an essential part of the RISC-loading mechanism [20]. Interestingly as discussed above, out of ten *Leishmania* proteins detected as a part of Ago-complexes, two were putative HSP70 and HSP70-raleted proteins. This raises the possibility that *Leishmania* HSP70 competes with host HSP70 for binding to the host Ago-complexes. In light of our recent finding regarding enrichment of sncRNAs and HSPs in the exosomes of *L*. *donovani* [30,57], it is tempting to speculate that *Leishmania* delivers its HSPs and sncRNAs to the host cell through exosomes, to skew the host RNAi by loading exogenous sncRNAs, and modulate the host gene expression in favour of parasite survival. Supporting this hypothesis is the observation that exosomes enriched in sncRNAs and proteins, are secreted in the cytosol of infected cell [30,58].

**Fig 7:**
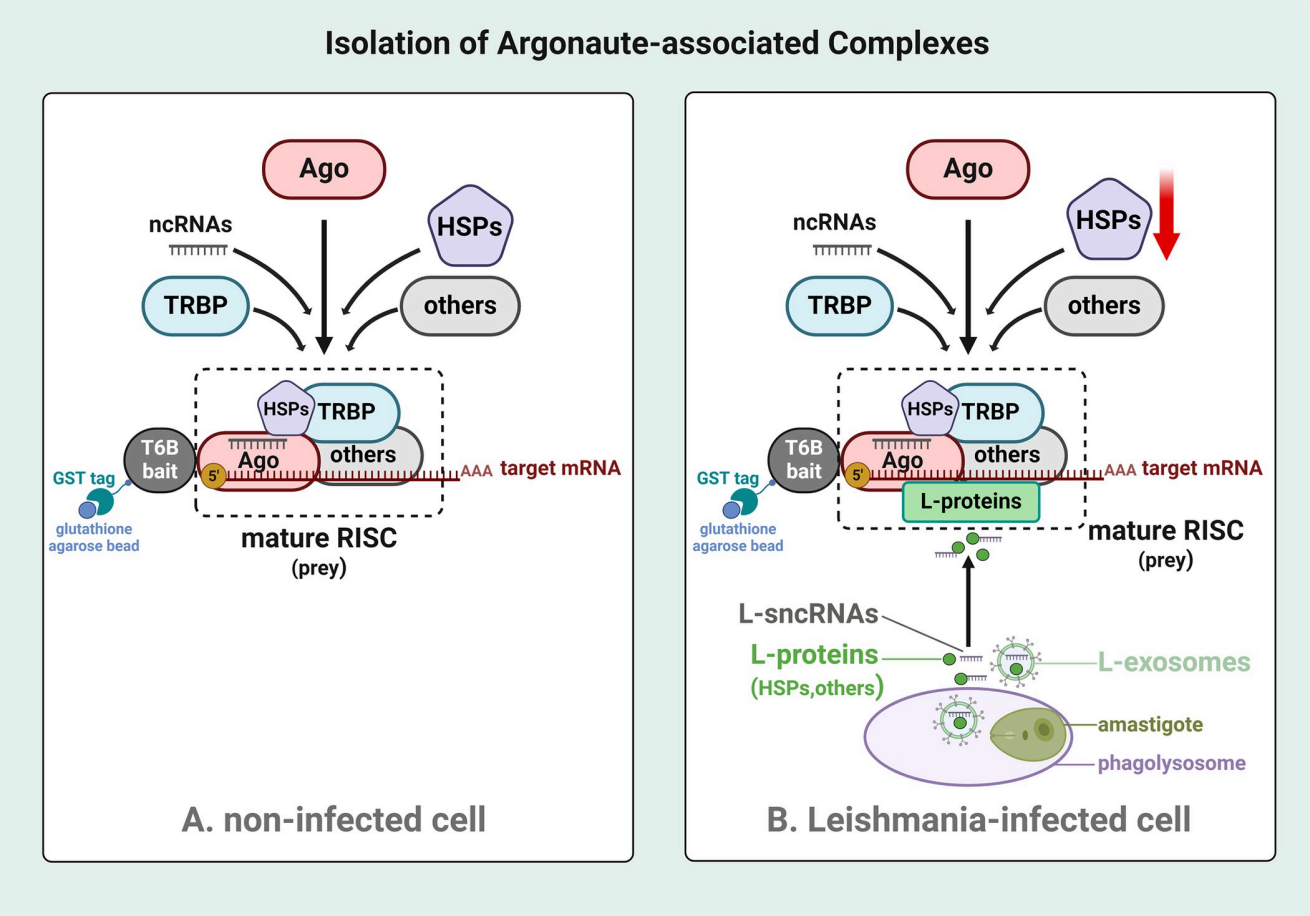
Schematic diagram depicting modulation of macrophage Ago-associated complexes by *Leishmania*. **(A)** RNAi is a post-transcriptional gene silencing pathway where sequence-specific sncRNA is able to silence genes via degradation/blocking translation of complementary mRNA. sncRNA is loaded into Ago protein containing RNA-induced silencing complex. In most situations, TNRC6 (a scaffolding protein) is recruited to bridge interaction between sncRNA, Ago and effector proteins to form an inhibitory silencing complex for gene regulation. **(B)** During infection, *Leishmania* proteins (L-proteins) and sncRNAs (L-sncRNAs) are released in the host cell’s cytoplasm via secretory exosomes from outside or inside *Leishmania*-containing phagolysosomes. L-sncRNAs are loaded into the host Ago proteins, mediated by L-HSPs, and form an effector complex to regulate host gene expression. The presence of *Leishmania* proteins in the host RISC suggests modulation of the composition of RISC during infection (Created with BioRender.com).

Within this context, it is established that sncRNAs are not confined to their point of origin within an organism. In fact, the bidirectional trafficking of sncRNAs between host and corresponding pathogen has been clearly shown in many studies [34,59–62]. This phenomenon known as cross-kingdom RNA interference, which operates as both a host’s defense mechanism and a strategy employed by pathogens to skew the host RNAi machinery to their advantage [60,61]. The hypothetical model depicting the possibility of cross-kingdom RNAi during *Leishmania* infection is presented as Fig 7.

A previous study has analyzed Ago1 and Ago2 interacting proteins using co-immunoprecipitation experiments followed by mass spectrometry [63]. As expected from this comprehensive proteomic analysis of the Ago1 and Ago2 interactomes, many proteins, including well-characterized Dicer and Mov10 proteins, were identified. In a recent study, Ago2 co-immunoprecipitated proteins from human red blood cells were subjected to proteome analysis. Unexpectedly but interestingly, this study did not identify Dicer or TRBP in Ago2 interactome [64]. Both these studies expected to pull down proteins interacting with sncRNA loaded Ago proteins, unoccupied Ago proteins, TNRC6 and its associated proteins. In addition, the co-immunoprecipitation assay used in these studies is also expected to pull Ago-interacting proteins involved in non-canonical functions of Ago proteins [51]. Nevertheless, from these studies, it will be challenging to identify proteins that selectively interact with Ago proteins, particularly in RISC.

The current study used GST-T6B pull-down assay to identify Ago-associated proteins in noninfected and *Leishmania-*infected macrophages. T6B peptide used for this assay is derived from the Ago-binding domain (ABD) of human TNRC6B-iso2 (80 amino acids between 559 and 996 amino acid sequence) [35]. This peptide has been shown to interact with all human Ago proteins with equal affinity [35]. All three TNRC6 paralogs share two distinct structural domains, the N-terminal Ago binding domain and the C-terminal/silencing domain. It is also known that TNRC6 binds to sncRNAs loaded Ago proteins with increased affinity [25]. T6B peptide was selected from the high Ago binding region of TNRC6. Thus, T6B peptide being part ABD domain, GST-T6B affinity was chosen to selectively isolate interacting proteins associated with functional Ago proteins, possibly involved in RNAi. Support for the absence of Dicer and Mov10 in our study comes from a relevant previous study where full-length TNRC6B was co-immunoprecipitated. Analysis of immunoprecipitated proteins revealed the absence of Dicer and Mov10 while successfully co-immunoprecipitating Ago1-4. On the other hand, the co-immunoprecipitation of Ago proteins pulled down Mov10 and Dicer [65]. Together, it shows that Dicer and Mov10 do not interact with TNRC6B. It seems reasonable to assume that Dicer–an enzyme that generates sncRNAs (21–23 nucleotides)–may not have a role to play once snRNAs are loaded into Ago proteins for subsequent RNAi.

Thus, use of GST-T6B affinity beads enabled the selective capture of Ago proteins and their associated proteins. Therefore, a limitation of the Ago-APP approach is the potential omission of proteins that interact with other domains like silencing domain of TNRC6. Thus, by using T6B peptide in Ago-APP, TNRC6 silencing domain-associated proteins were not detected.

While this study provides an atlas of Ago-associated proteins (Table 1), it is likely not complete. This protocol is unable to detect Ago-associated proteins that are not expressed in dTHP-1 cells, inactive under our experimental conditions, in undetectable quantity, or detached from the Ago-complexes during the isolation procedure. Nevertheless, the proteome of Ago-associated proteins isolated from *Leishmania-*infected host cells is comprised of proteins enriched in RNAi-related activities. We note that this study is restricted to characterize the protein composition of host RISC complex in response to *Leishmania* infection. However, it could open the way for more in-depth studies of the role of RNAi in *Leishmania* pathogenesis and may also have implications for other intracellular pathogens.

In addition, our work using human macrophage-*Leishmania* as a model host-pathogen interaction system is also an important contribution since most of prior understanding of the role of RNA interference mechanisms in infection is based on plants and insects. This work is, to the best of our knowledge, the first to analyze *Leishmania-*mediated alterations in host RNAi complexes. The presence of *Leishmania* proteins in host Ago-complexes is suggestive of a smart strategy used by *Leishmania* to hijack the effector complex of host RNAi. An in depth understanding of cross-kingdom RNAi in context of *Leishmania* infection could provide novel therapeutic strategies for managing and treating leishmaniasis.

## Supporting information

S1 Fig Schematic diagram of metabolic SILAC quantitative proteomics of Ago-complexes. THP-1 cells were cultured in light and medium SILAC media. Labeled THP-1 cells were differentiated with PMA and subsequently incubated with *L*. *donovani* for 24 hours. Then, the cytoplasmic fractions from non-infected (NI) and infected cells were precleared with GST (control) beads followed by incubation with GST-T6B affinity beads. Released interacting proteins were mixed in a 1:1 ratio and used for mass spectrometry, allowing for a quantitative comparison between non-infected and infected cells.
(TIFF)

S2 Fig Silver staining of proteins present in eluted materials from Ago-APP. The cytoplasmic fractions from non-infected and *Leishmania*-infected macrophages were subjected to Ago-APP affinity column as described in “Materials and method”. Bound materials were concentrated and separated on 8% SDS-PAGE and proteins were visualized by silver staining. (NI: non-infected, I: *Leishmania*-infected cells).
(TIFF)

S1 Table (XLSX)

S1 Raw images (PDF)
