# Supplementary figures and images for: Characterization of Argonaute-containing protein complexes in Leishmania-infected human macrophages
Source: PLoS One. 2024 May 23;19(5):e0303686. doi: 10.1371/journal.pone.0303686 (PMC11115314; doi:10.1371/journal.pone.0303686)

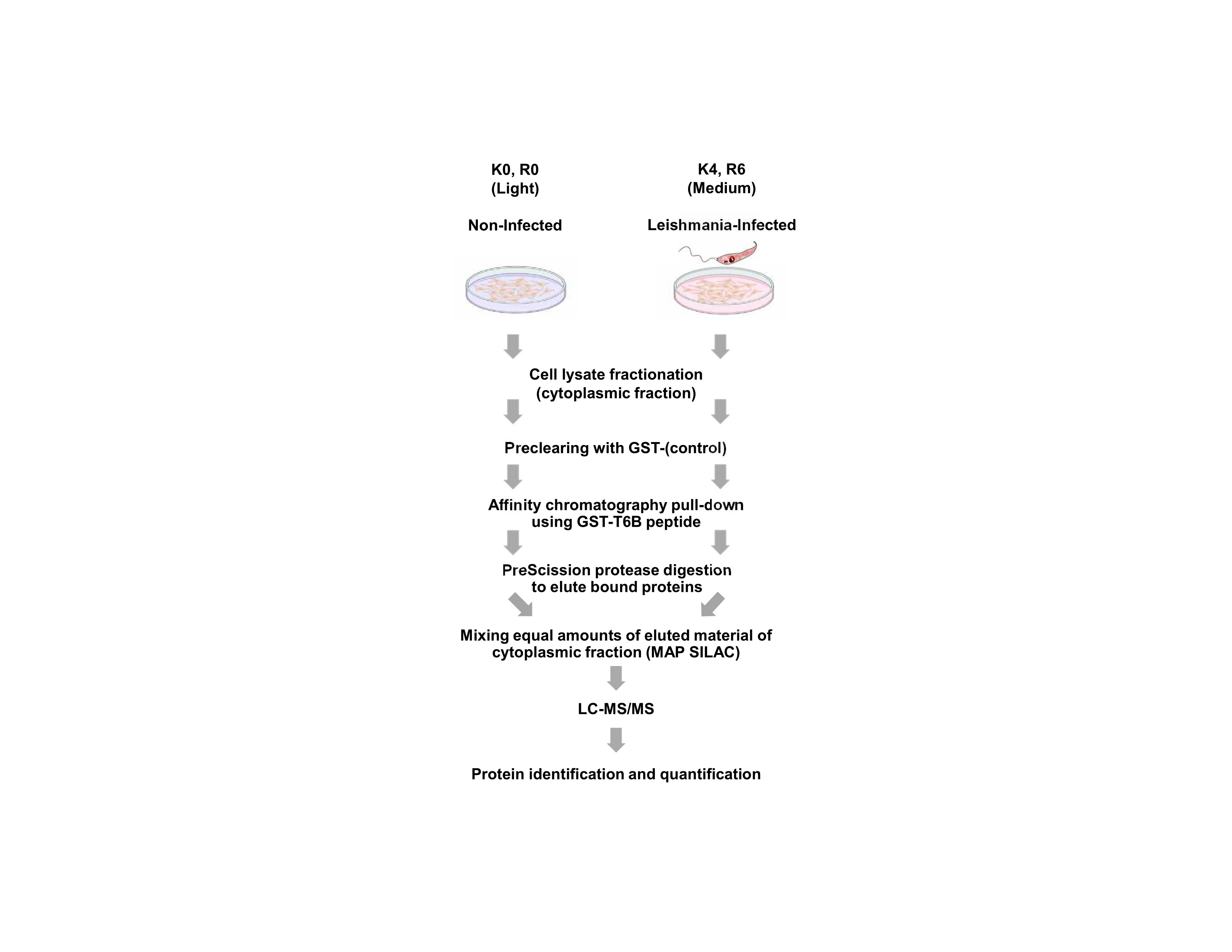

Supplement: S1 Fig — THP-1 cells were cultured in light and medium SILAC media. Labeled THP-1 cells were differentiated with PMA and subsequently incubated with L. donovani for 24 hours. Then, the cytoplasmic fractions from non-infected (NI) and infected cells were precleared with GST (control) beads followed by incubation with GST-T6B affinity beads. Released interacting proteins were mixed in a 1:1 ratio and used for mass spectrometry, allowing for a quantitative comparison between non-infected and infected cells. (TIFF) [file pone.0303686.s001.tiff]

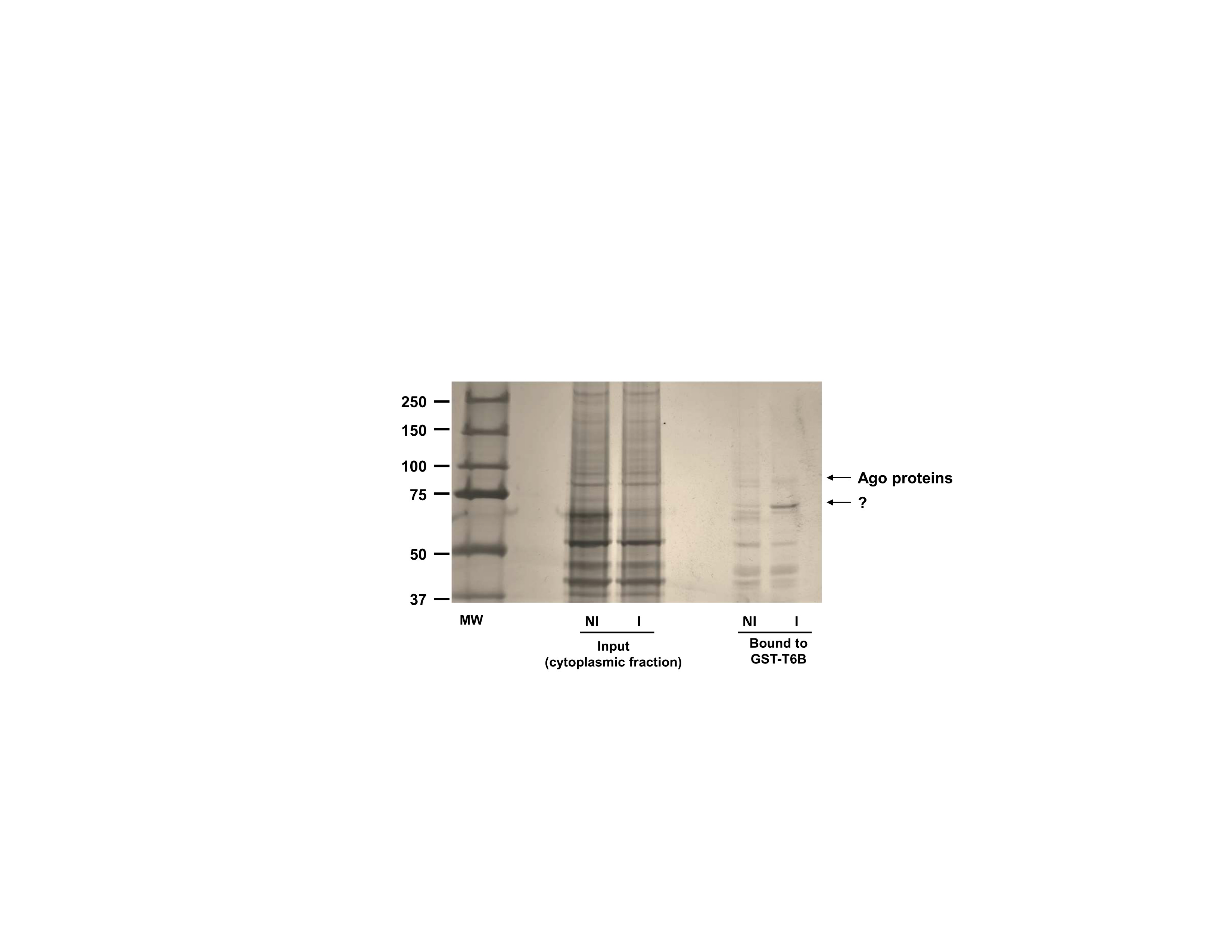

Supplement: S2 Fig — The cytoplasmic fractions from non-infected and Leishmania-infected macrophages were subjected to Ago-APP affinity column as described in “Materials and method”. Bound materials were concentrated and separated on 8% SDS-PAGE and proteins were visualized by silver staining. (NI: non-infected, I: Leishmania-infected cells). (TIFF) [file pone.0303686.s002.tiff]
