# Supplementary material for: Characterization of Argonaute-containing protein complexes in Leishmania-infected human macrophages
Source: PLoS One. 2024 May 23;19(5):e0303686. doi: 10.1371/journal.pone.0303686 (PMC11115314; doi:10.1371/journal.pone.0303686)
Supplement: S1 Raw images — (PDF) [file pone.0303686.s004.pdf]

Fig 1A. Western blot to evaluate the specificity of the T6B affinity beads, non-specific controls (Actin and GAPDH)
8% Tris-Glycine SDS-PAGE
Nitrocellulose

Image captured via ECL and X-ray film

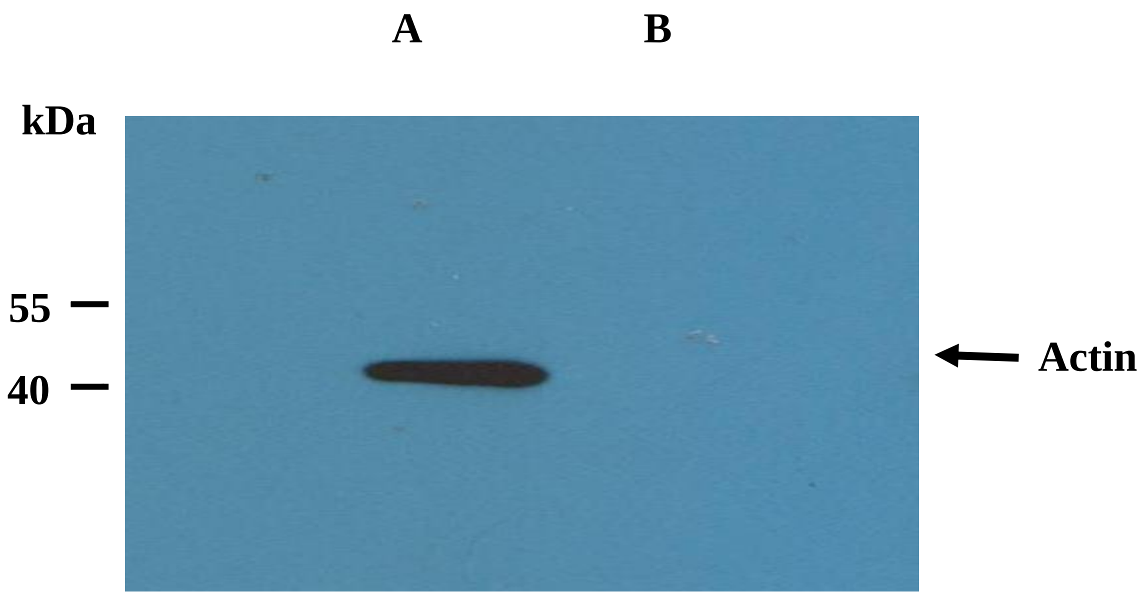

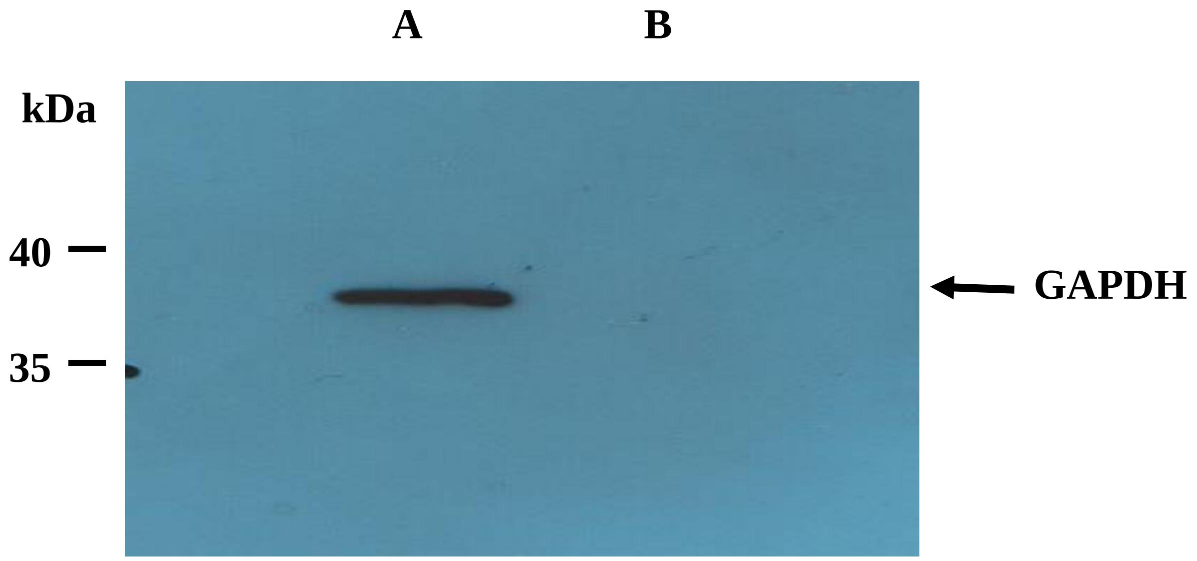

A. Input: total cell lysate of differentiated THP-1 (dTHP-1) cells

B. Total cell lysate of dTHP-1 cells, bound to GST-T6B

Fig 1B. Western blot to evaluate the specificity of the T6B affinity beads, positive controls (Argonaute proteins)
8% Tris-Glycine SDS-PAGE
Nitrocellulose

Image captured via ECL and X-ray film

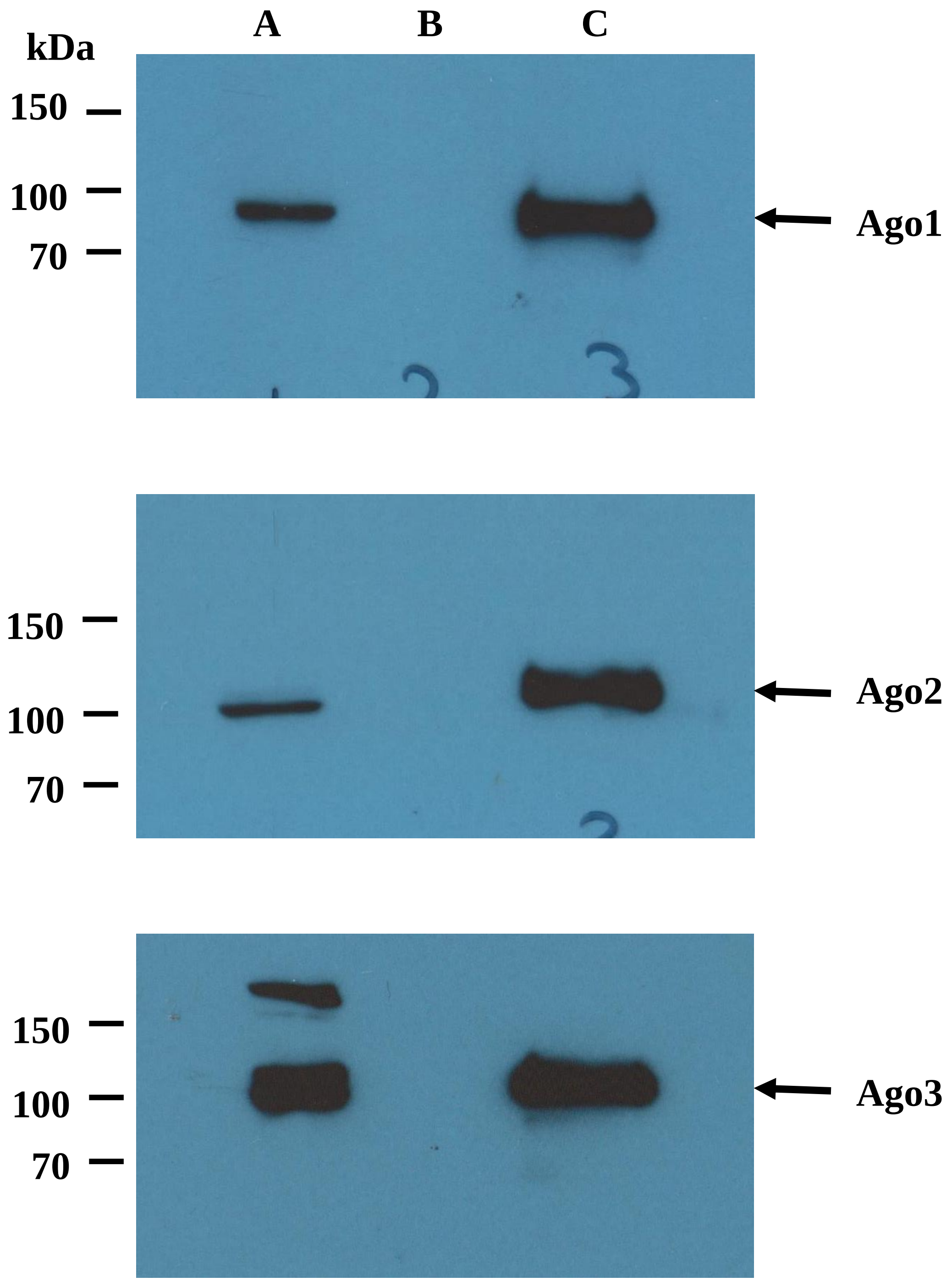

A. Input: total cell lysate of dTHP-1 cells
B. Total cell lysate of dTHP-1 cells, bound to GST (control)
C. Total cell lysate of dTHP-1 cells bound to GST-T6B

Fig 1C. Northern blot to evaluate the specificity of the T6B affinity beads, let7a-5p miRNA
15% polyacrylamide TBE- urea (7M) gel
Hybond N+ nylon membrane

Image captured via ECL and X-ray film

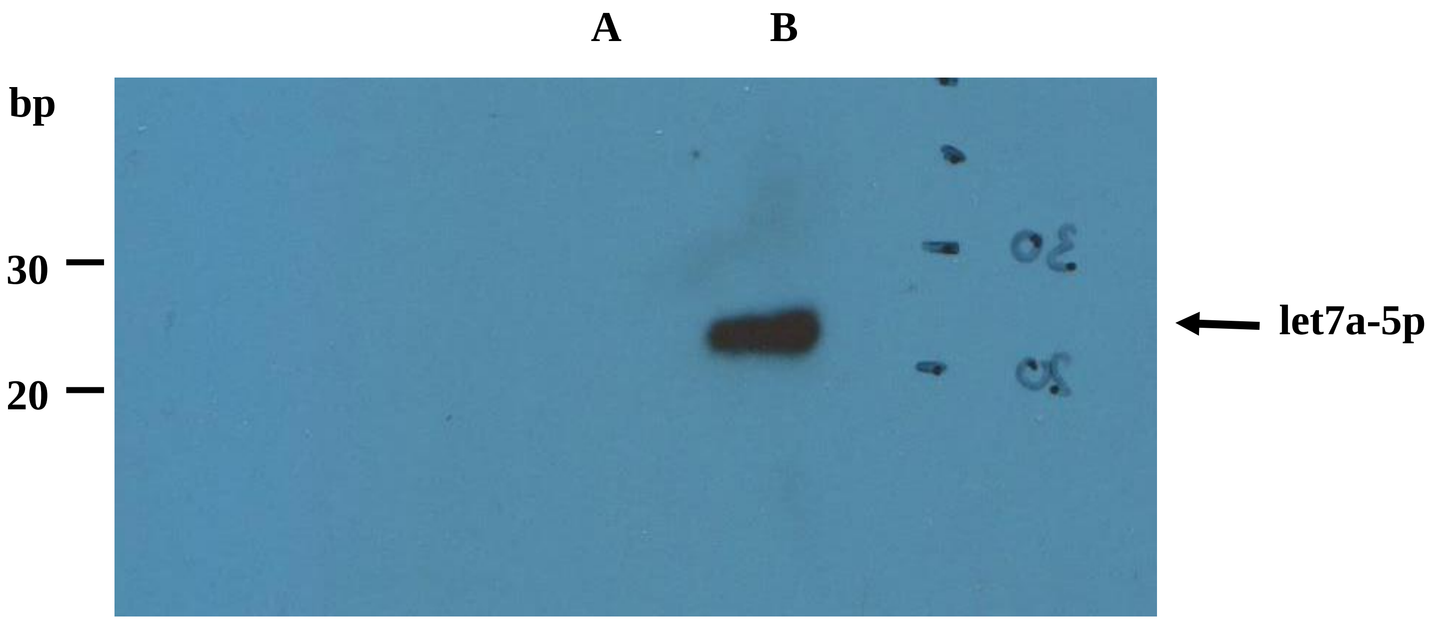

A. dTHP-1 cells total RNA, bound to GST (control)
B. dTHP-1 cells total RNA, bound to GST-T6B

Fig 2A. Western blot to evaluate the purity of the cytoplasmic and ER-free nuclear fractions using fraction-specific markers
8% Tris-Glycine SDS-PAGE
Nitrocellulose

Image captured via ECL and X-ray film

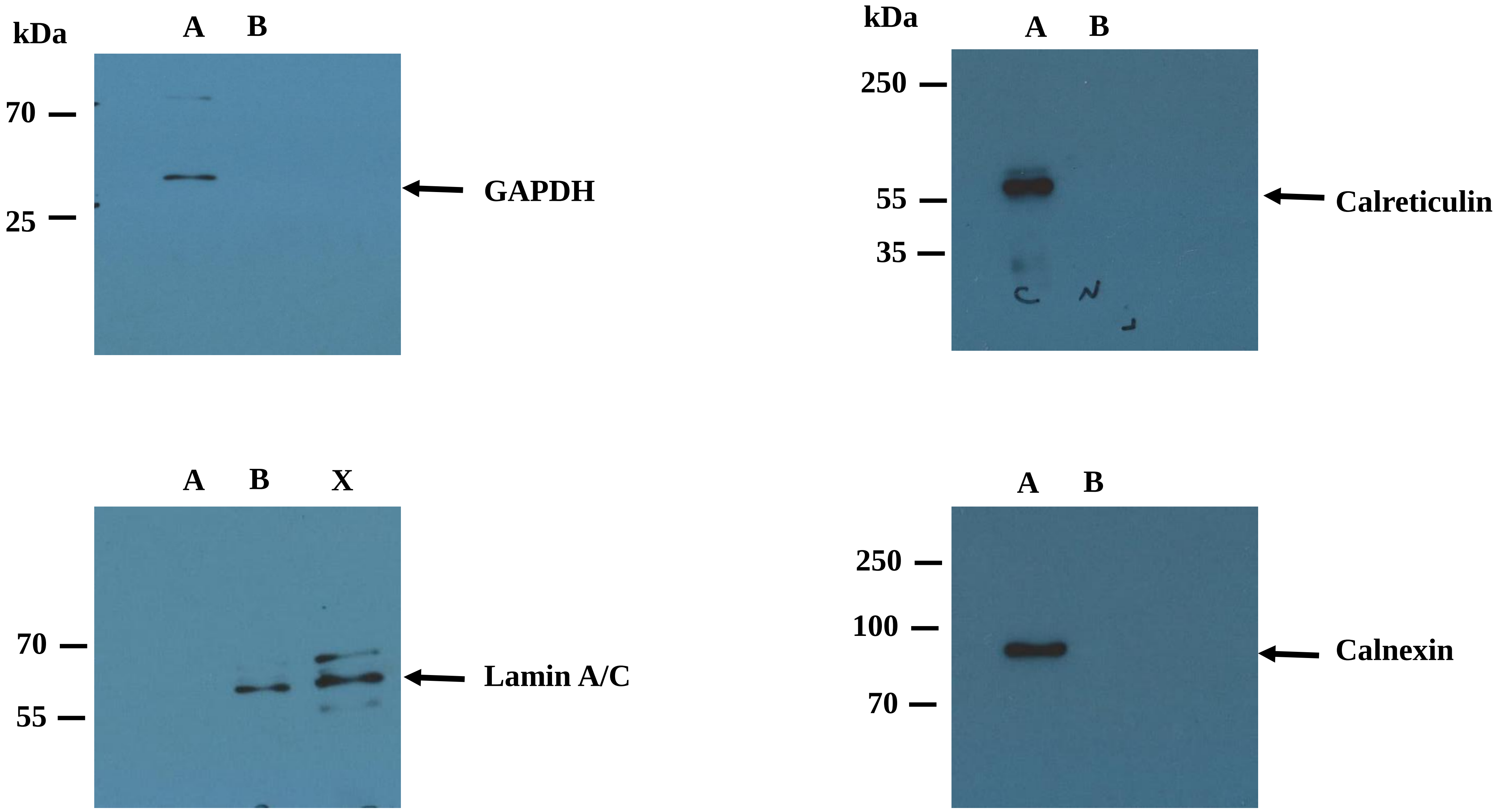

A. Cytoplasmic fraction of dTHP-1 cells, bound to GST-T6B
B. Nuclear fraction of dTHP-1 cells, bound to GST-T6B

Fig 2B. Western blot to evaluate subcellular localization of Argonaute proteins and TRBP
8% Tris-Glycine SDS-PAGE
Nitrocellulose

Image captured via ECL and X-ray film

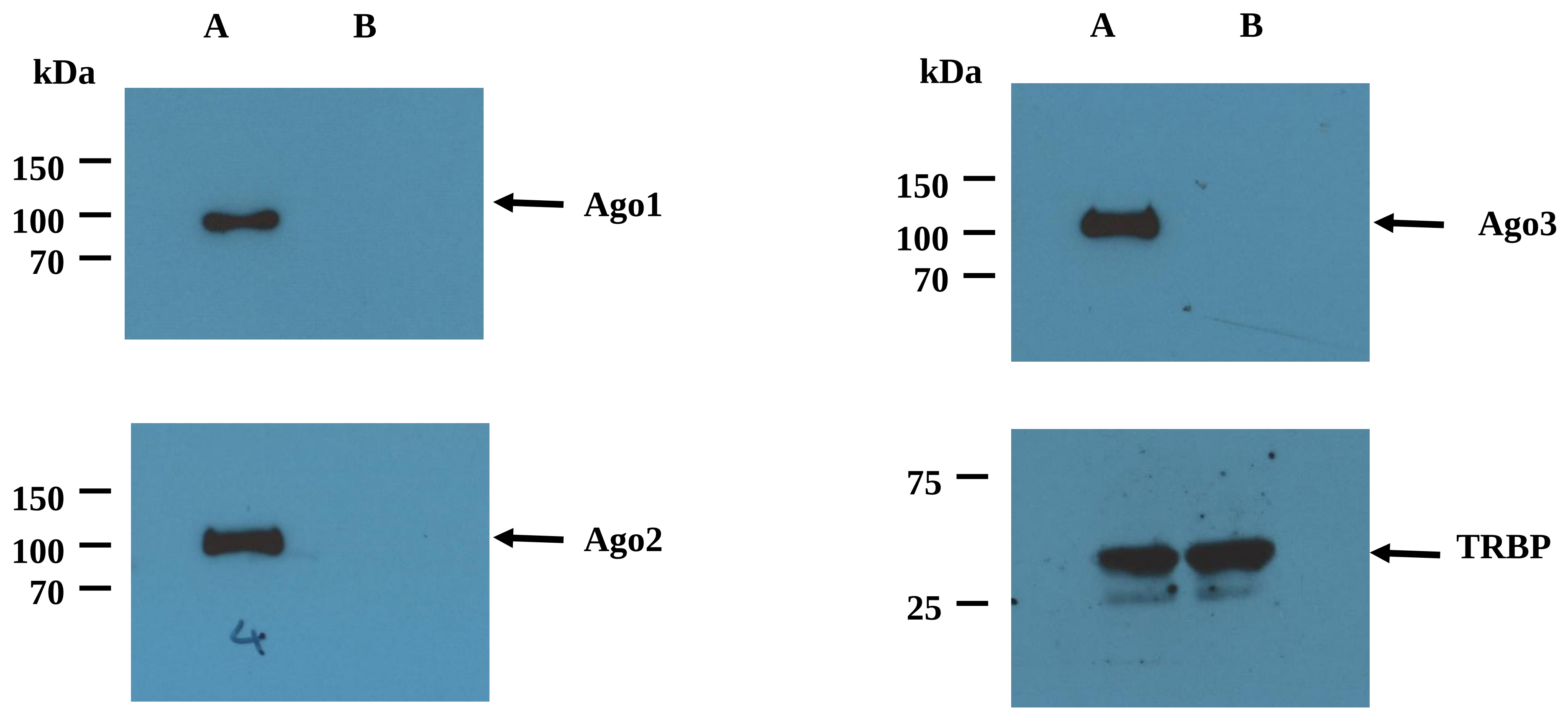

A. Cytoplasmic fraction of dTHP-1 cells, bound to GST-T6B
B. Nuclear fraction of dTHP-1 cells, bound to GST-T6B

Fig 6B. Western blot to evaluate direct interaction of *Leishmania* HSP70 and T6B affinity beads
8% Tris-Glycine SDS-PAGE
Nitrocellulose

Image captured via ECL and X-ray film

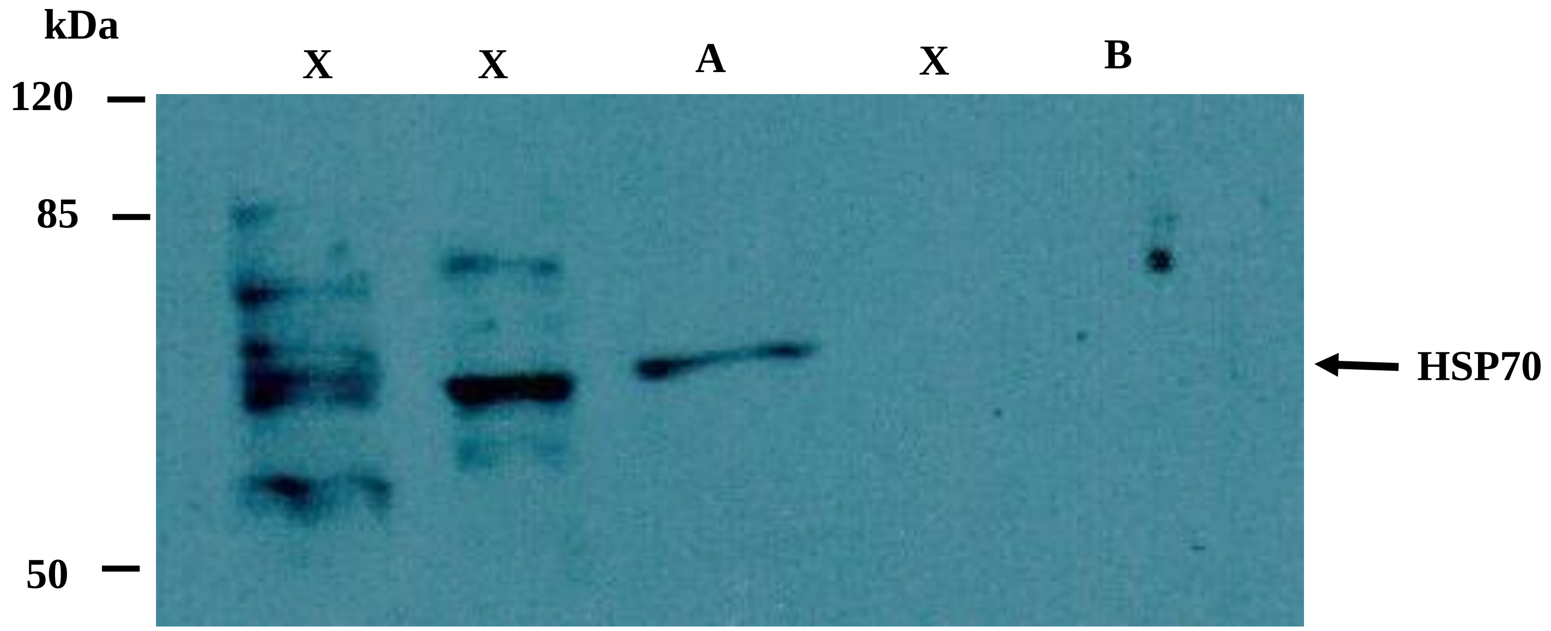

A. Input: *L. donovani* total cell lysate
B. *L. donovani* total cell lysate, bound to GST-T6B

S2 Fig. Western blot followed by silver staining to qualify eluted proteins via Ago-APP
8% Tris-Glycine SDS-PAGE

Image captured using a mobile device

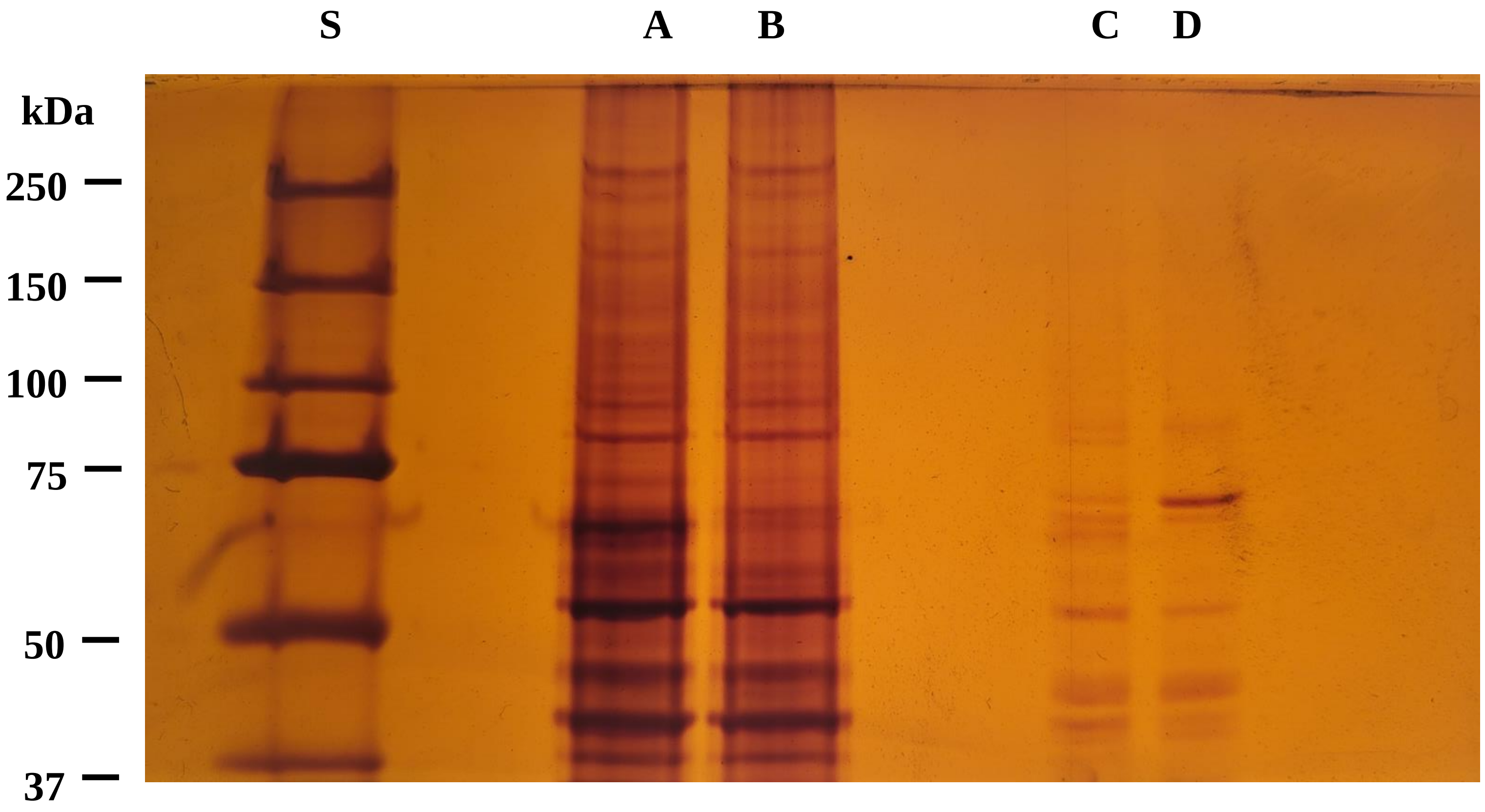

S: Standard
A. Input: cytoplasmic fraction of dTHP-1 cells
B. Input: cytoplasmic fraction of *Leishmania*-infected dTHP-1 cells
C. Cytoplasmic fraction of dTHP-1 cells , bound to GST-T6B
D. Cytoplasmic fraction of *Leishmania*-infected dTHP-1 cells, bound to GST-T6B
